# Unraveling the multi-targeted curative potential of bioactive molecules against cervical cancer through integrated omics and systems pharmacology approach

**DOI:** 10.1038/s41598-022-18358-7

**Published:** 2022-08-21

**Authors:** Murali Aarthy, Pandiyan Muthuramalingam, Manikandan Ramesh, Sanjeev Kumar Singh

**Affiliations:** 1 Computer Aided Drug Design and Molecular Modeling Lab, Department of Bioinformatics, Alagappa University, Karaikudi, Tamil Nadu 630003 India; 2 Department of Biotechnology, Science Campus, Alagappa University, Karaikudi, Tamil Nadu 630003 India

**Keywords:** Computational biology and bioinformatics, Systems biology, Bioinformatics

## Abstract

Molecular level understanding on the role of viral infections causing cervical cancer is highly essential for therapeutic development. In these instances, systems pharmacology along with multi omics approach helps in unraveling the multi-targeted mechanisms of novel biologically active compounds to combat cervical cancer. The immuno-transcriptomic dataset of healthy and infected cervical cancer patients was retrieved from the array express. Further, the phytocompounds from medicinal plants were collected from the literature. Network Analyst 3.0 has been used to identify the immune genes around 384 which are differentially expressed and responsible for cervical cancer. Among the 87 compounds reported in plants for treating cervical cancer, only 79 compounds were targeting the identified immune genes of cervical cancer. The significant genes responsible for the domination in cervical cancer are identified in this study. The virogenomic signatures observed from cervical cancer caused by E7 oncoproteins serve as the potential therapeutic targets whereas, the identified compounds can act as anti-HPV drug deliveries. In future, the exploratory rationale of the acquired results will be useful in optimizing small molecules which can be a viable drug candidate.

## Introduction

In cancer biology, viruses possess a foremost role over the past two decades, and especially, the tumor viruses encompassing RNA and DNA with the fundamental contributions are highly responsible^1^. Numerous oncogenes carried by retroviruses that were derived from the cellular genes are involved in the signaling and control of cell growth. These oncogenes from the viral origin are requisite for the replication and cell transformation^2^. The organization of the genome in retroviruses differentiates the representations of the simple and complex viruses. Retroviruses which does not possess viral oncogenes like avian leukosis virus and mouse mammary tumor virus induce tumors in animals^2^. Further, the virus which is responsible for the development of malignancies with expanded latency in relation towards the environmental and host associated cooperating events exists. The oncogenicity of the virus and the mode of infection discriminate the nature from other carcinogenic agents. Better insights on the pathogenesis of viral infection and host responses are very important in understanding the cancers in detail. Oncogenic viruses belong to diverse families and employ varied mechanism for the development of cancer^3^. Martin and Gutkind states that the Hepatitis B virus (HBV), Human T-cell lymphotropic virus (HTLV), Epstein-Barr virus (EBV), Human papillomavirus (HPV), Hepatitis C virus (HCV), and Kaposi’s associated sarcoma virus (KSHV) contributes towards 15% of the human cancer^4^.

Italian physicist Ciuffo identified the etiology of warts in human around 1907 and identified the link with HPV in the 1970s. The infections caused by HPV in the cervix lead to cervical malignancy and other related warts. These viruses are non-enveloped double-stranded DNA viruses constituting triple segments namely the late, early and genomic regions^5^. The most common second malignant tumour that threatens the health of female all over the world is cervical cancer. The persistent infection caused by the human papillomavirus is the necessary cause of cervical cancer^6^. Further, different types of HPV have been characterized based on the ability to promote the proliferation of infected cells leading to cell transformation as low, intermediate and high-risk human papillomaviruses^3,7^. The HPV types 11, 6, 42, 40, 44, 43, 54, and 53 are intended to be low risk due to their involvement in the formation of benign warts whereas the HPV types 31, 35, 33 and 52 are identified to be the intermediate risk since it is responsible for mild and severe lesions. The HPV types 16, 18, 39 and 45 cause malignant transformation through their directing features towards the tumor suppressor proteins^8,9^. These viruses infect the keratinocyte, germ layers of the mucous membranes and skin. Also, the HPV infection is comparable among the different tissues which contaminate the basal layer of the cervix and enters the tonsillar epithelium divulging the crypt cells^10,11^. The genome of HPV is circular with 8 kbp which encodes major proteins namely the E1, E2, E4, E5, E6, E7, L1 and L2 in the early and late region. The proteins in the early region are responsible for the replication and transcription of genome and apoptosis control, immune modulation and modification in the structure whereas the proteins in the late region are responsible for the development of capsid proteins^12^. The early proteins E6 and E7 were observed to be oncogenic which depicts the major role in cell line transformation and regulation of the stability in chromosomes. These oncoproteins were responsible for their association with the tumor suppressors p53 and pRB proteins which are involved in the inactivation of the suppression ability of tumor leading to the formation of cancer^12,13^. E7 oncoprotein is strongly necessary for the lifecycle of virus and the development of cancer from the benign stage to invasive cancer. This oncoprotein E7 acts as the regulator of transcription and also suppresses numerous other genes which are regulated through the interferon and Nuclear Factor Kappa B (NF-kB) pathways which results in enabling the evasion of the immunity^14^. HPV E7 is termed to be the phosphoprotein whereas the process of phosphorylation is thought to be important for the function and regulation^15^.

In general, it is clearly evident that the treatment for cancer involves surgery, radiotherapy and chemotherapy depending on the phase and position of the tumor. Numerous anti-cancer drugs affect the normal cells that are divided rapidly under normal circumstances and cause serious side effects^16^. Hence, the identification of potent anticancer agents with fewer side effects is highly essential in the recent period. It is evident from the studies that the phytochemicals obtained from the ethnobotanically active plants that hinders the carcinogenic processes and will be useful for the treatment of cancer with minimal side effects^17^. They are classified into phenols, terpenoids, alkaloids and flavonols. Also, they possess beneficial roles like prevention of cancer, diabetes, antiviral and antimicrobial activities^18^. Generally, phytochemicals were in use from the ancient period by millions of people which arbitrate its positive health benefits by disturbing the affected molecular targets like genes^19^.

International Union for Conservation of Nature (IUCN) and the World Wildlife Fund has reported the availability of 80,000 flowering plant species for medicinal purposes^20^. *Cremanthodium humile, Zingiber officinale, Cordyceps pruinosa, Ficus hirta, Mangifera indica, Nigella sativa, Corallina pilulifera, Citrus grandis, Cassia tora, Crocus sativa, Pinus massoniana, Pinellia pedatisecta, Dutchesnea indica, Solanum nigrum, Triticum aestivum, Cinnamomum cassia, Artemisia afra, Argimonia eupatoria, Pterocarpus santalinus* and *Phaseolus vulgaris* are some of the essential medicinal plants in Indian traditional medicines. These plants possess various medicinal activities and especially they were reported to treat cervical cancer^21–30^. Despite the noteworthy role of phytochemicals from the ethnobotanical plants, the mechanism of action and their momentous immune-responsive human targets that involve various biological function, need to be elucidated strongly.

Our study endeavored to identify the activity of bioactive compounds and the mechanism of immunological features from the plant species *M. indica, N. sativa, Z. officinale, C.grandis, Ziziphus jujube, Z. mauritiana* and *C.cassia* against the cervical cancer. The involvement of immune- responsive genes which could be the reason for developing cervical cancer with their molecular cross-talks can be explored in this holistic study. Along with this, we attempted to explore the closely related genes with cervical cancer and the phytochemicals for the accomplishment of immunobiological activity. Advancements in the systems pharmacology and analytical tools like immuno-transcriptomics and interactome analysis help us to disclose the molecular interactions of phytochemicals for the treatment of harmful infection by viruses like HPV. Thus, our study divulges information concerning the immunological mechanism of phytochemicals and their pharmacological properties. The profiling with the immuno-transcriptomic data uncovers the differentially expressed genes associated with cervical cancer. Subsequently novel bioactive compounds with indispensable pharmacological activities are filtered out and these compounds that directly targets the human immune responsive genes of different functions are identified. Further, the immunological targets of cancer were then introduced to some specific database to discover the immunological mechanisms and the signaling pathways of bioactive compounds from the ethnobotanical plants. We assure that these explorations of the immunological mechanisms of the bioactive compounds will extensively endorse the expansion of novel drugs for the treatment of cervical cancer and other related cancers in near future.

## Materials and methods

The systems pharmacology and the multi-omics approaches have been incorporated together to unravel the significant curative efficacy of potential therapeutic phytocompounds from natural plants to combat cervical cancer caused by HPV. These approaches consist of target mining and functional enrichment analysis to identify the phytocompounds used in the treatment of cervical cancer caused by HPV. Further, the systemic network construction and analysis to demonstrate the molecular machinery of phytocompounds extracted from the medicinal plants in treating cervical cancer is characterized in this pilot study. Further, the gene ontology and STRING analysis for HPV immune-responsive genes paves way for the diverse biological pathway analysis. This also helps in reveling the functional mode of key players in multiple nodes from the immunological pathway level^19^.

### Mining of immune responsive genes of human transcriptome related to cervical cancer

The human transcriptomic datasets of cervical cancer cases were collected from the Array express database (https://www.ebi.ac.uk/arrayexpress/) with the ID: E-GEOD-39001 and E-GEOD-46842^31^ which encodes the affymetrix data. This data has been manually curated using excel Microsoft. Upon completion of the curation, the transcriptomic datasets was then imported into the Network Analyst 3.0. database (https://www.networkanalyst.ca/) especially to the Gene expression table to check the total number of genes^32^. The dataset is generally saved in the .txt file format which is plotted in the excel file with the microarray data intensities corresponding to the immune responsive genes of healthy controls and cancer patients. These data are depicted in the time series of columns and rows in the excel data format as sample and class. Followed by the incorporation of the dataset, normalization and filtering is carried out for making certain that the distribution of expression is comparable across the inclusive experiments and to remove the inconsistent data, respectively. Further, the differential gene expression analysis is carried out to witness the significant immune genes which are responsive through the Limma statistical model with the adjusted *P*-value less than 0.05 along with the representation of 1.0 as Log2 fold change value. The identified genes were further studied for the over representation analysis (ORA) functional enrichment along with the tissue-specific interactions through the inbuilt databases in Network Analyst 3.0. In order to make the results more clear, the probe set ID has been referred through the BioGPS database (http://biogps.org/dataset/) and the gene name was confirmed^32^ and provided in Supplementary Table S1.

### Pharmacologically active phytocompounds

Exhaustive information on the pharmacologically active molecules from the plants *M. indica, N. sativa, Z. officinale, C. grandis, Z. jujube, Z. mauritiana* and *C. cassia* were retrieved from the web sources and literature^22–30,33^. List of the active compounds from the natural medicinal plants with their abbreviations were given in Table 1. The information like the canonical SMILES of the compounds was retrieved from the PubChem database (https://pubchem.ncbi.nlm.nih.gov/)^34^ are also provided in Table 1. The identified compounds from the medicinal plants were searched against the *Homo sapiens* in SwissTargetPrediction tool (http://swisstargetprediction.ch/index.php) in order to retrieve the human targets especially the immune responsive genes^35^.

**Table 1:** List of Compounds with its Abbreviations & SMILES.

S. no.	Name of the compound	Abbreviation	SMILES
*M. indica*			
1.	Friedelin	FD	CC1C(=O)CCC2C1(CCC3C2(CCC4(C3(CCC5(C4CC(CC5)(C)C)C)C)C)C)C
2.	Humulene	HL	CC1=CCC(C=CCC(=CCC1)C)(C)C
3.	Elemene	EL	CC(=C)C1CCC(C(C1)C(=C)C)(C)C=C
4.	Epigallocatechin gallate	EGCG	C1C(C(OC2=CC(=CC(=C21)O)O)C3=CC(=C(C(=C3)O)O)O)OC(=O)C4=CC(=C(C(=C4)O)O)O
5.	Isomangiferin	IM	C1=C2C(=CC(=C1O)O)OC3=C(C2=O)C(=CC(=C3C4C(C(C(C(O4)CO)O)O)O)O)O
6.	Linalool	LL	CC(=CCCC(C)(C=C)O)C
7.	β—Carotene	β-C	CC1=C(C(CCC1)(C)C)C=CC(=CC=CC(=CC=CC=C(C)C=CC=C(C)C=CC2=C(CCCC2(C)C)C)C)C
8.	β—sitosterol	β-S	CCC(CCC(C)C1CCC2C1(CCC3C2CC=C4C3(CCC(C4)O)C)C)C(C)C
9.	Octylgallate	OG	CCCCCCCCOC(=O)C1=CC(=C(C(=C1)O)O)O
10.	Linolenic acid	LA	CCC=CCC=CCC=CCCCCCCCC(=O)O
11.	Methyl gallate	MG	COC(=O)C1=CC(=C(C(=C1)O)O)O
12.	Ocimene	OI	CC(=CCC=C(C)C=C)C
13.	Kaempferol	KP	C1=CC(=CC=C1C2=C(C(=O)C3=C(C=C(C=C3O2)O)O)O)O
*N. sativa*			
14.	Thymoquinone	TQ	CC1=CC(=O)C(=CC1=O)C(C)C
15.	Alpha hederin	AH	CC1C(C(C(C(O1)OC2C(C(COC2OC3CCC4(C(C3(C)CO)CCC5(C4CC=C6C5(CCC7(C6CC(CC7)(C)C)C(=O)O)C)C)C)O)O)O)O)O
16.	Nigellicine	NC	CC1=CC(=O)C2=C(N3CCCCN3C2=C1)C(=O)O
17.	Nigellidine	ND	CC1=CC(=O)C2=C(N3CCCCN3C2=C1)C4=CC=C(C=C4)O
18.	Thymohydroquinone	THQ	CC1=CC(=C(C=C1O)C(C)C)O
19.	Carvacrol	CC	CC1=C(C=C(C=C1)C(C)C)O
20.	Carvone	CV	CC1=CCC(CC1=O)C(=C)C
21.	Thymol	TL	CC1=CC(=C(C=C1)C(C)C)O
22.	Limonene	LN	CC1=CCC(CC1)C(=C)C
23.	4-Terpineol	4-TR	CC1=CCC(CC1)(C(C)C)O
24.	Alpha-pinene	α-PN	CC1=CCC2CC1C2(C)C
25.	Tricyclene	TRC	CC1(C2CC3C1(C3C2)C)C
26.	Camphene	CP	CC1(C2CCC(C2)C1=C)C
27.	Sabinene		CC(C)C12CCC(=C)C1C2
28.	1,8-Cineole		CC1(C2CCC(O1)(C(C2)OC3C(C(C(C(O3)CO)O)O)O)C)C
29.	Alpha—Terpinene	α-TPN	CC1=CC=C(CC1)C(C)C
30.	Borneol	BR	CC1(C2CCC1(C(C2)O)C)C
31.	Pinocarvone	PCV	CC1(C2CC1C(=C)C(=O)C2)C
32.	Cyclosativene	CS	CC(C)C1CCC2(C3C1C4C2(C4C3)C)C
33.	Alpha-Longicyclene	α-LC	
34.	Alpha-Copaene	α-CN	CC1=CCC2C3C1C2(CCC3C(C)C)C
35.	Alpha—Longifolene	α-LN	CC1(CCCC2(C3C1C(C2=C)CC3)C)C
36.	Palmitic acid	PA	CCCCCCCCCCCCCCCC(=O)O
37.	Octadecanoic acid	ODC	CCCCCCCCCCCCCCCCCC(=O)O
*Z. officinale*			
38.	6-Gingerol	6-GL	CCCCCC(CC(=O)CCC1=CC(=C(C=C1)O)OC)O
39.	6-Shogaol	6-SL	CCCCCC=CC(=O)CCC1=CC(=C(C=C1)O)OC
40.	6-paradol	6-PL	CCCCCCCC(=O)CCC1=CC(=C(C=C1)O)OC
41.	10-gingerol	10-GL	CCCCCCCCCC(CC(=O)CCC1=CC(=C(C=C1)O)OC)O
42.	10-Shogaol	10-SL	CCCCCCCCCC=CC(=O)CCC1=CC(=C(C=C1)O)OC
43.	6-dehydroshogaol	6-DHSL	CCCCCC=CC(=O)C=CC1=CC(=C(C=C1)O)OC
44.	Gingerenone	GRN	COC1=C(C=CC(=C1)CCC=CC(=O)CCC2=CC(=C(C=C2)O)OC)O
*C. grandis*			
45.	Naringin	NN	CC1C(C(C(C(O1)OC2C(C(C(OC2OC3=CC(=C4C(=O)CC(OC4=C3)C5=CC=C(C=C5)O)O)CO)O)O)O)O)O
46.	Nobiletin	NT	COC1=C(C=C(C=C1)C2=CC(=O)C3=C(O2)C(=C(C(=C3OC)OC)OC)OC)OC
47.	Tangeretin	TT	COC1=CC=C(C=C1)C2=CC(=O)C3=C(O2)C(=C(C(=C3OC)OC)OC)OC
48.	5-Demethyltangeretin	5-DMTT	COC1=CC=C(C=C1)C2=CC(=O)C3=C(C(=C(C(=C3O2)OC)OC)OC)O
49.	Sinensetin	SNT	COC1=C(C=C(C=C1)C2=CC(=O)C3=C(C(=C(C=C3O2)OC)OC)OC)OC
50.	Naringenin	NGN	C1C(OC2=CC(=CC(=C2C1=O)O)O)C3=CC=C(C=C3)O
51.	Hesperidin	HD	CC1C(C(C(C(O1)OCC2C(C(C(C(O2)OC3=CC(=C4C(=O)CC(OC4=C3)C5=CC(=C(C=C5)OC)O)O)O)O)O)O)O)O
52.	Methoxylated	MOX	CCCCCCCCCCOC
*Z. jujube*			
53.	Ursolic acid	UA	CC1CCC2(CCC3(C(=CCC4C3(CCC5C4(CCC(C5(C)C)O)C)C)C2C1C)C)C(=O)O
54.	Oleanolic acid	OA	CC1(CCC2(CCC3(C(=CCC4C3(CCC5C4(CCC(C5(C)C)O)C)C)C2C1)C)C(=O)O)C
55.	Betulinic acid	BA	CC(=C)C1CCC2(C1C3CCC4C5(CCC(C(C5CCC4(C3(CC2)C)C)(C)C)O)C)C(=O)O
*Z. Mauritiana*			
56.	Glaucine	GC	CN1CCC2=CC(=C(C3=C2C1CC4=CC(=C(C=C43)OC)OC)OC)OC
*C. cassia*			
57.	Cinnamaldehyde	CDY	C1=CC=C(C=C1)C=CC=O
58.	Cinnamic acid	CNA	C1=CC=C(C=C1)C=CC(=O)O
59.	Cinnamyl acetate	CLA	CC(=O)OCC=CC1=CC=CC=C1
60.	-Thujene	TJ	CC1C=CC2(C1C2)C(C)C
61.	-Terpineol	TPL	CC1=CCC(CC1)C(C)(C)O
62.	-Cubebene	CBB	CC1CCC(C2C13C2C(=C)CC3)C(C)C
63.	Eugenol	EGL	COC1=C(C=CC(=C1)CC=C)O
64.	-Caryophyllene	CPL	CC1=CCCC(=C)C2CC(C2CC1)(C)C
65.	Terpinolene	TRPL	CC1=CCC(=C(C)C)CC1
66.	E-Nerolidol	E-NL	CC(=CCCC(=CCCC(C)(C=C)O)C)C
67.	L-Borneol	L-BL	CC1(C2CCC1(C(C2)O)C)C
68.	Caryophyllene Oxide	CPLO	CC1(CC2C1CCC3(C(O3)CCC2=C)C)C
69.	Coumarin	CM	C1=CC=C2C(=C1)C=CC(=O)O2
70.	Myrcene	MYE	CC(=CCCC(=C)C=C)C
71.	α -Phellandrene	α-PDR	CC1=CCC(C=C1)C(C)C
72.	Terpinolene	TRPL	CC1=CCC(=C(C)C)CC1
73.	Isoborneol	IBL	CC1(C2CCC1(C(C2)O)C)C
74.	Geraniol	GL	CC(=CCCC(=CCO)C)C
75.	Safrole	SFL	C=CCC1=CC2=C(C=C1)OCO2
76.	Phenylacetaldehyde	PADY	C1=CC=C(C=C1)CC=O
77.	Vanillin	VL	COC1=C(C=CC(=C1)C=O)O
78.	Salicylaldehyde	SADY	C1=CC=C(C(=C1)C=O)O
79.	Acetophenone	APE	CC(=O)C1=CC=CC=C1
80.	Anisaldehyde	ASDY	COC1=CC=C(C=C1)C=O
81.	β -Bisobolol	β-BL	CC1=CCC(CC1)(C(C)CCC=C(C)C)O
82.	α -Muurolol	α-ML	CC1=CCC(CC1)(C(C)CCC=C(C)C)O
83.	Patchoulene	PCL	CC1CCC2=C1CC3CCC2(C3(C)C)C
84.	Guaicol	GL	COC1=CC=CC=C1O
85.	Methyl alaninate	MAT	CC(C(=O)OC)N
86.	Undecanoic acid	UDCA	CCCCCCCCCCC(=O)O
87.	Decanoic acid	DCA	CCCCCCCCCC(=O)O

### Compound target network (CTN) construction and features of human targets

The construction of the CTN illuminates the multi-target therapeutic features of the pharmacologically active plant compounds. In this construction, we observed, the interaction between the human immune-responsive genes and the phytocompounds representing the interconnection which is visualized through the Cytoscape *v*3.8.0. In the obtained interactome, the node depicts the compounds and targets whereas the edge denotes the molecular interaction between the compounds and targets^36^. The significant 35 immune responsive genes/targets obtained from the literature and CTN analysis were used for the retrieval of molecular features like official Gene Symbol with the name, position of the target, chromosome numbers and orthologs of the differentially expressed immune responsive genes from the NCBI-gene database (https://www.ncbi.nlm.nih.gov/gene) and The Human Protein Atlas (https://www.proteinatlas.org/)^19,37^.

### Pharmacological features of the compounds

The compounds with the respective canonical SMILES were subjected to the Molinspiration online tool (https://molinspiration.com/) in order to obtain significant molecular features of the phytocompounds. Along with this the bioactive score for the vital targets such as the GPCR ligand activity, protease inhibitor activity (Pi), Kinase Inhibitor activity (Ki) and number of violations (nVio) were predicted^38–40^.

### Gene ontology enrichment analysis

The genes which are differentially expressed with the encoding gene symbols were uploaded to the GOnet (https://tools.dice-database.org/GOnet/) and Metascape (https://metascape.org/gp/index.html#/main/step1) databases in order to attain the ontology against the humans with the significant threshold of *q*-value which is greater than 0.05. The immune-responsive genes were pigeonholed with the molecular function and biological process based on the functional enrichment classification of the database^35^.

### Construction of protein–protein interaction (PPI) Network associated with HPV 16 E7 and other cancer associated proteins

The cellular machinery of the proteins is formed based on the interactions made by the proteins. Recognition of the PPI perseveres to be one of the foremost determinations in modern biology as well as the improvement of protein therapeutics^41^. In our study, we attempted to identify the interactions between the oncoprotein of E7 and the dominated therapeutic proteins of human. The STRING database (https://string-db.org/) is used for the identification of the human proteins interacting with the obtained immune-responsive genes by providing the input manually obtained from the CTN^42^. The STRING database depicts the interactions between proteins which compasses direct interaction physically and the indirect correlation representing the functional aspects of the protein^43^. The information obtained from the database regarding the PPI is curated from the experimental data, prediction from the genomic features, text mining from the scientific articles and from various database^44^. This database provides the score based on the weight and impact of the interactions^45^. The PPI network of E7 oncoprotein of HPV with the confidence score of greater or equal to 0.7 was retrieved from STRING Viruses *v*10.5 (http://viruses.string-db.org/). These proteins interacting with E7 were imported to the cytoscape *v*3.8.0 software for better visualization^36^. The STRING Viruses generated the PPI network with the query as E7 oncoprotein in the database providing the direct interactions with the imputed protein, consisting of all the proteins and interactions between them^43^.

## Results

### Immune responsive genes from Meta-analysis of human immunotranscritpome

**Figure 1:**
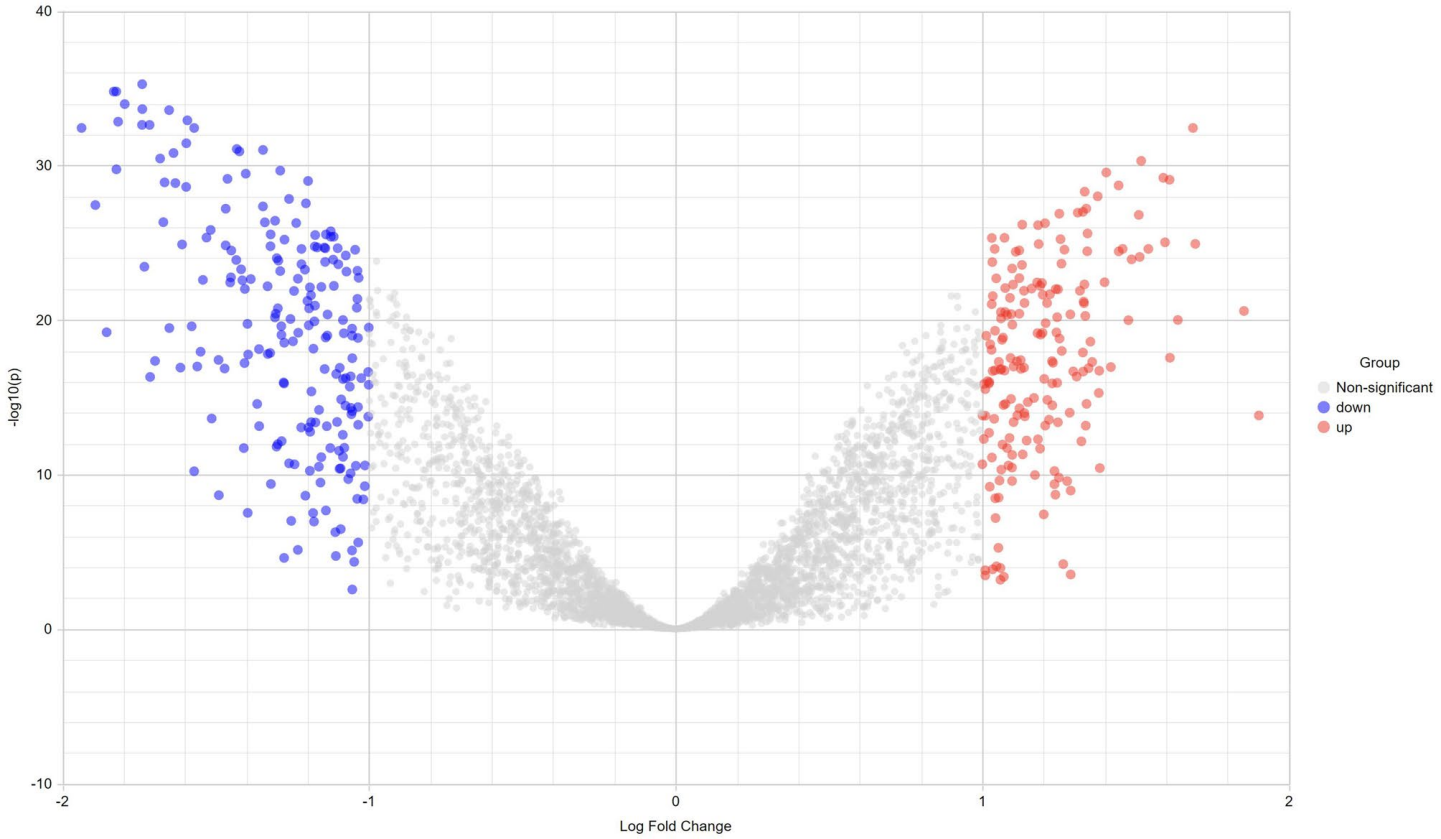
Volcano plot of the differential gene expression of immune genes of human. The scattered points indicate the up and down regulated and non-significant genes based on the threshold applied. Red spheres represent up regulation, blue spheres represent the down regulation and black spheres represent non-significant genes.

**Figure 2:**
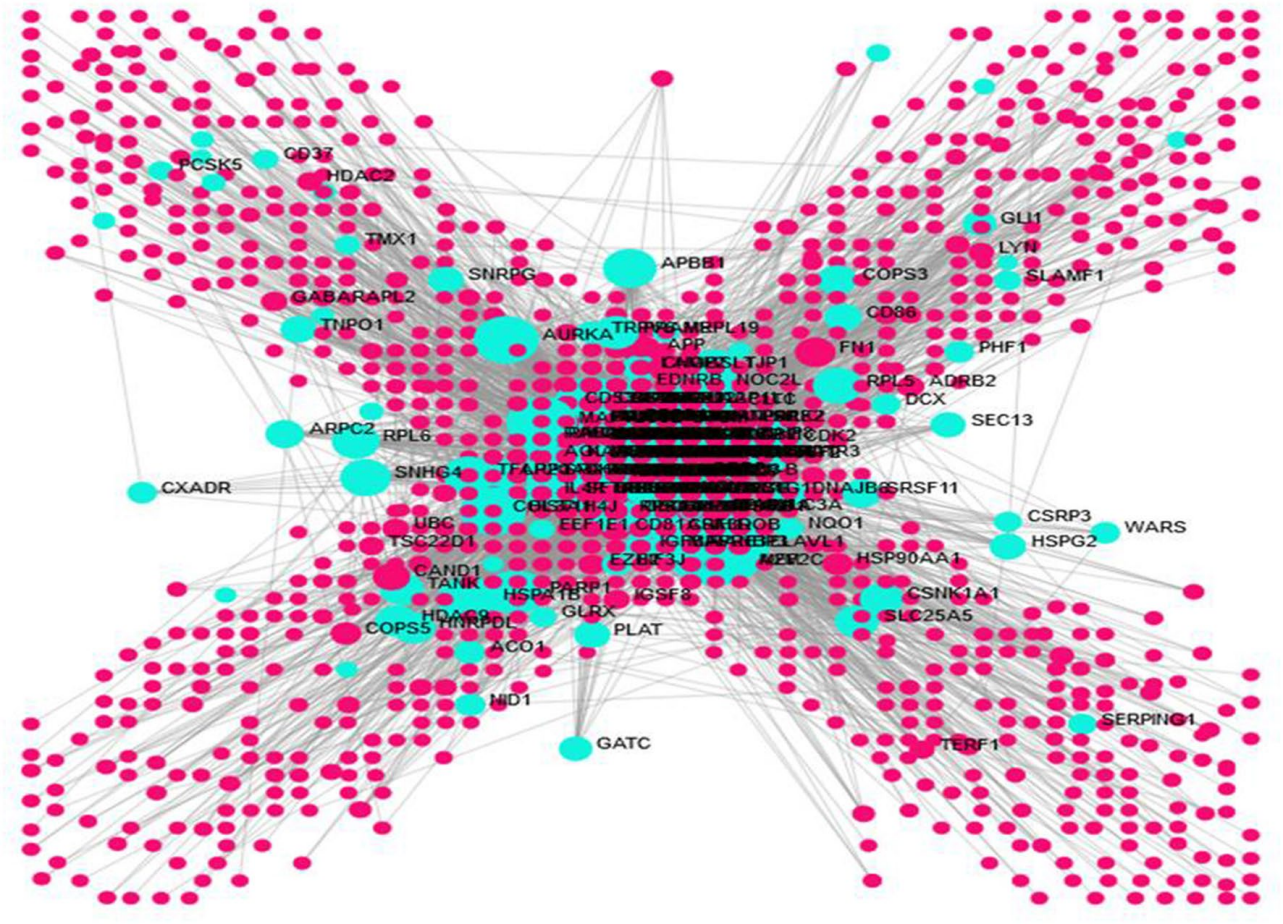
Tissue-specific PPI network for the differentially expressed genes. The green color represents the differentially expressed genes and the pink color spheres represent the interaction with various human immune responsive genes.

**Figure 3:**
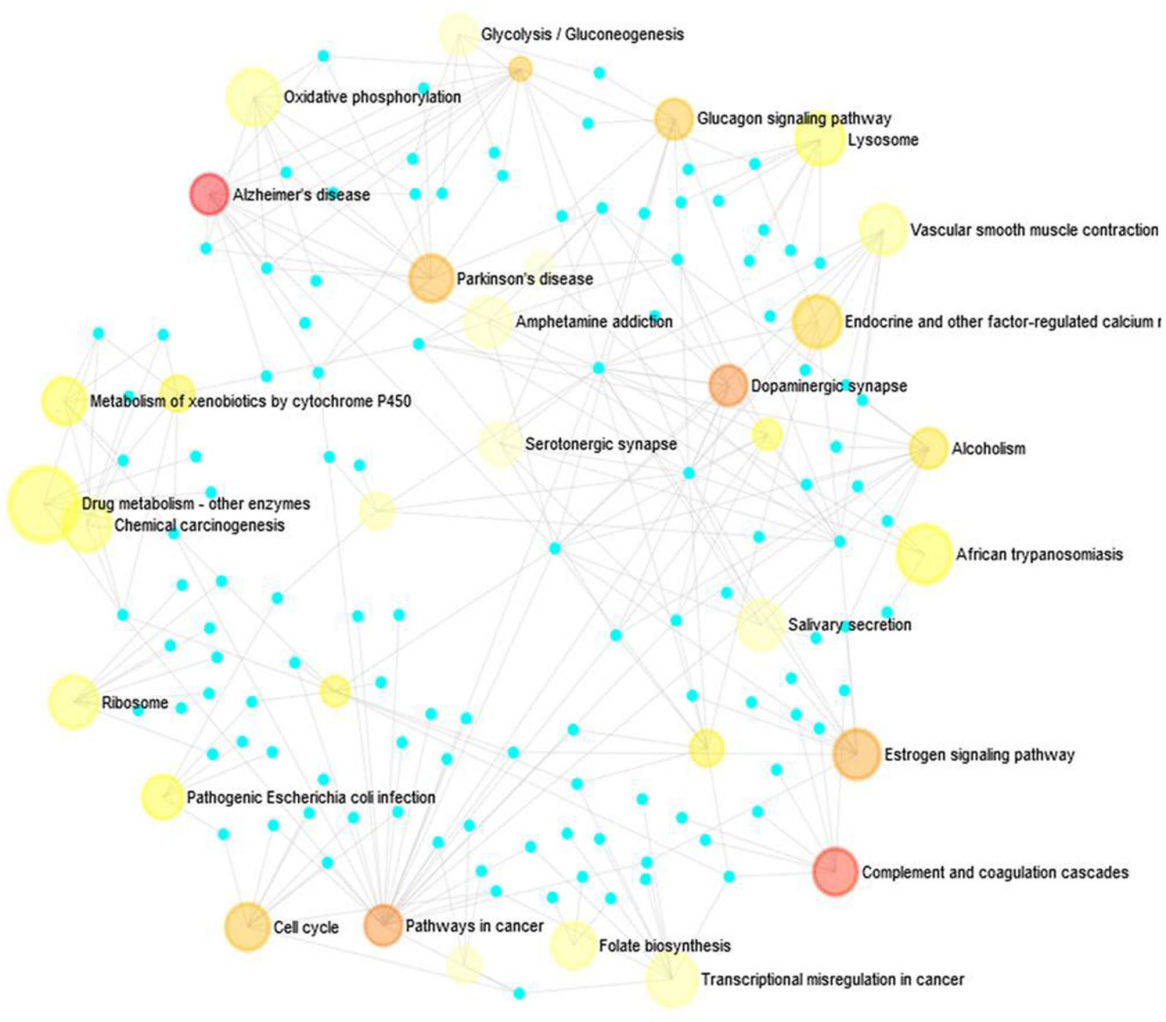
Enrichment network analysis observed through Network Analyst tool representing the pathways involved in differentially expressed genes.

The transcriptomics dataset contains 8353 immune responsive genes which are diversified into 59 samples out of which 3680 genes were commonly found in the meta differential expression depicted in Fig. 1. Figure 1 states that, immune responsive genes that are up, down and non-significantly expressed. Followed by the interactive volcano plot, heat map profiling revealed that 384 immune responsive genes were expressed differentially in cervical cancer cases during various time periods when compared with the healthy controls which are clearly evident in Supplementary Fig. 1. The genes that are up and down regulated among the 384 genes were listed in Supplementary Table S2. The differentially expressed genes are visualized through the tissue specific PPI in the tissue type whole blood represented in Fig. 2. This network encompasses 4184 nodes and 8064 edges. Further, the pathway-based ORA enrichment network showed involvement in various biological pathways which is represented in Fig. 3.

### Retrieval of information regarding phytocompounds

A total of 87 molecules were retrieved through the literature and employed as a query in the PubChem database to fetch the canonical SMILES which are represented in Table 1 along with the abbreviations. The abbreviations mentioned were further used during the CTN construction. The obtained information about the compound was used further for the biomolecular analyses.

### Pharmacologically active compounds and its interaction with human targets

The active phytocompounds that direct towards the human immune receptors were computed through the SwissTargetPrediction tool. Among the 87 compounds, 79 compounds were appreciably targeting 35 out of the 384 human immune-responsive and literature-retrieved receptors/genes that are differentially expressed between healthy and cancer cases. The comprehensive list of the phytocompounds and the equivalent interaction with the human immune responsive genes are provided in Table 2.

**Table 2:** Compounds and the respective human immune genes.

S. no.	Compound name	Human immune genes
*M. indica*		
1.	Friedelin	SERPINA6
2.	Humulene	GLI1
3.	Elemene	MAOA
4.	Epigallocatechin gallate	MMP2, MMP12, AURKA
5.	Isomangeferin	SLC29A1, MMP2
6.	Linalool	PARP1
7.	Β—Carotene	RARA
8.	Β—Sitosterol	SREBF2
9.	Octyl gallate	MAOA, CDK2, MMP12, ADORA2B, GSK3A, SREBF2
10.	Linoleneic acid	SERPINA6, PLA2GIB, MMP2
11.	Methylgallate	LDHA
12.	Ocimene	–
13.	Kaempferol	CYP1B1, PLA2GIB, PARP1
*N. .sativa*		
14.	Thymoquinone	MCL1, MAOA
15.	Alpha Hedirin	MMP2, AURKA, MMP12
16.	Nigellicine	MCL1
17.	Nigellidine	G6PC, CTSS, GSK3A
18.	Thymohydroquinone	PARP1, MMP2, LDHA, DHFR
19.	Carvacrol	AURKA
20.	Carvone	SERPINA6, PARP, NQO1, MAOA, CTSS
21.	Thymol	AURKA, CDK2, PLA2GIB, MAOA
22.	Limonene	SREBF2, SERPINA6, GLI1, PLA2GIB
23.	4-Terpineol	SREBF2, PLA2GIB, ADORA2B
24.	Alpha-pinene	SREBF2, PLA2GIB, RARA
25.	Tricyclene	–
26.	Camphene	SREBF2,
27.	Sabinene	–
28.	1,8-Cineole	–
29.	Alpha—Terpinene	RARA
30.	Borneol	DPP4, ICMT, SERPINA6, ADORA2B
31.	Pinocarvone	SERPINA6, PARP1, PLA2GIB
32.	Cyclosativene	–
33.	Alpha-Longicyclene	–
34.	Alpha-Copaene	SREBF2, SERPINA6, PLA2GIB,
35.	Alpha—Longifolene	–
36.	Palmitic acid	SERPINA6
37.	Octadecanoic acid	SERPINA6, MMP2, MCL1
*Z. officinale*		
38.	6-Gingerol	CDK2, MAOA, PARP1, GSK3A, NCOR2, PDE2A
39.	6-Shagaol	NCOR2, MMP12, PDE2A, PARP1, CDK2, SREBF2
40.	6-Paragaol	MAOA, PARP1, ADORA2B, SREBF2, NCOR2,
41.	10-Gingerol	CDK2, AURKA, MAOA, GSK3A,
42.	10-Shogaol	HDAC9, ADORA2B, MMP12, CDK2, SLC8A1, AURKA, DHFR,
43.	6-dehydroshogaol	MAOA, CDK2, AURKA, MMP12, DRD4, PARP1, NCOR2, CYP, SREBF2, PDE2A, HDAC9
44.	Gingerenone	AURKA, HDAC9, MMP12, DHFR, CDK2
*C. grandis*		
45.	Naringin	MMP12, CYP1B1, PARP1, ADORA2B, SLC29A1, CDK2, PLA2GIB
46.	Nobiletin	CYP1B1, PARP1, GSK3B, CDK2, MMP2, MAOA
47.	Tangeretin	CYP1B1, MCL1, PARP1, MMP2, MAOA
48.	5-Demethyltangeretin	MCL1, CYP1B1, MMP2, MAOA, MMP12,
49.	Sinensetin	PARP1, MMP2, MAOA, MMP12
50.	Naringenin	CYP1B1, PLA2GIB, MMP12, MMP2, CDK2, AURKA, MAOA
51.	Hesperidin	CYP1B1, MMP12, ADORA2B, SLC29A1, DHFR,
52.	Methoxylated	ICMT, PARP1, GRIA2, MAOA, PDE2A,
*Z. jujube*		
53.	Ursolic acid	PLA2GIB, SERPINA6, MMP2, SREBF2, EDNRB
54.	Oleanolic acid	PLA2GIB, SERPINA6, MMP2
55.	Betulinic acid	PLA2GIB, SERPINA6, MMP2,
*Z. .Mauritiana*		
56.	Glaucine	PTPRCAP, AURKA, PDE2A, CDK2,
*C. cassia*		
57.	Cinnamaldehyde	PARP1, MCL1, MAOA, CTSS,
58.	Cinnamic acid	MMP2, RNPEP, MAOA, CPA1, MCL1,
59.	Cinnamyl acetate	PARP1, CDK2,
60.	-Thujene	SREBF2, SERPINA6, PLA2GIB
61.	-Terpineol	SREBF2, PLA2GIB, PARP1, CTSS,
62.	-Cubebene	–
63.	Eugenol	PARP1, AURKA, CDK2, GSK3A,
64.	-Caryophyllene	GLI1, SREBF2, SERPINA6, RARA
65.	Terpinolene	SREBF2,
66.	E-Nerolidol	PDE2A, PARP1,
67.	L-Borneol	DPP4, ICMT, SERPINA6, ADORA2B,
68.	Caryophyllene Oxide	ICMT, PARP1,
69.	Coumarin	MAOA, NQO1, CDK2, AURKA, CYP1B1,
70.	Myrcene	GLI1,
71.	Alpha-Phellandrene	RARA, GLI1,
72.	Terpinolene	GLI1, SREBF2,
73.	Isoborneol	DPP4, ICMT, SERPINA6, ADORA2B,
74.	Geraniol	GSK3B, ICMT, GRIA2, PDE2A, MAOA,
75.	Safrole	NQO1, ADORA2B, CDK2, PARP1
76.	Phenylacetaldehyde	MCL1, PARP1, HDAC9, CTSS, CDK2,
77.	Vanillin	MMP2, MCL1, PARP1, MMP12, GSK3A,
78.	Salicylaldehyde	PARP1, LDHA, GRIA2, GSK3A, MMP2, DPP4,
79.	Acetophenone	MCL1, PARP1,
80.	Anisaldehyde	MCL1, PARP1, CTSS
81.	Beta-Bisobolol	SREBF2, SERPINA6, ICMT, PDE2A, PARP1, PLA2G1B,
82.	Alpha-Muurolol	SREBF2, SERPINA6, ICMT, PDE2A, PARP1, PLA2G1B,
83.	Patchoulene	SREBF2, SERPINA6, PLA2G1B,
84.	Guaicol	NQO1, MAOA, PARP1, GSK3A, CDK2, ADORA2B, CDK2,
85.	Methyl alaninate	DPP4
86.	Undecanoic acid	SERPINA6, RARA, SLC16A1, MMP2, MCL1, MMP12,
87.	Decanoic Acid	SERPINA6, RARA, SLC16A1, CPT1A, MMP2, MCL1,

### CTN analysis

**Figure 4:**
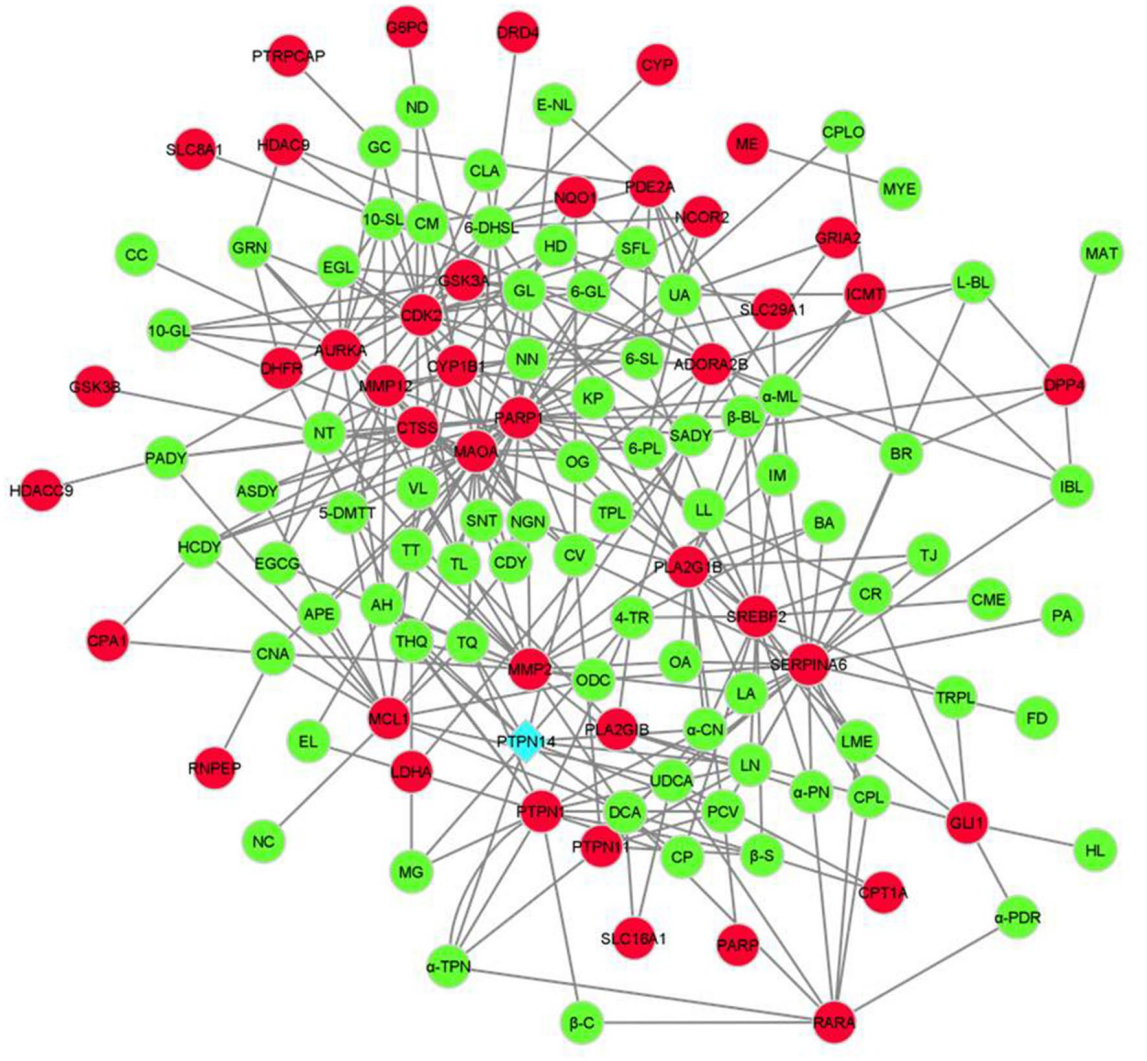
Visualization of CTN. Red color represents the immune responsive genes and green color represents the compound and blue color represents the tumor suppressor gene targeted by HPV.

The CTN based cross talks between the 79 active compounds and 35 significant immune-responsive genes were depicted in Fig. 4. This analysis discloses the multi target strategy which is noteworthy feature of phytocompounds. The detailed information on the connection between the phytocompounds and the immune-responsive genes revealed the therapeutic ability of the bioactive molecules present in the Indian herbal plants such as *M. indica, N. sativa, Z. officinale, C. grandis, Z. jujube, Z. mauritiana* and *C. cassia* for combating cervical cancer caused by HPV infection through the transduction and modulating the signals of possible immune responsive genes.

### Properties of the human immune responsive genes

A total of 35 immune responsive genes have been targeted by 79 phytocompounds. The corresponding information on the immune receptors namely chromosome number, the full name of the genes, physical position and the orthologs details were obtained and provided in Table 3. This information paves way for the delineation of the detailed molecular function. Further, the pictorial representation of the significant gene and its involvement in various biological pathways were presented in Supplementary Fig. S2.

**Table 3:** Molecular attributes of the identified thirty-five human immune responsive genes.

S. no.	Gene symbol	Gene name	Chromosome number	Start	End
1.	SERPINA6	Serpin family A member 6	14	94174322	94390654
2.	GLI1	GLI family zinc finger 1	12	57459785	57472451
3.	MAOA	Monoamine oxidase A	X	43655006	43746817
4.	MMP12	Matrix metallopeptidase 12	11	102862736	102874982
5.	AURKA	Aurora kinase A	20	56369390	56392308
6.	SLC29A1	Solute carrier family 29 member 1	6	44219587	44234144
7.	PARP1	Poly(ADP-ribose) polymerase 1	1	226360691	226408093
8.	RARA	Retinoic acid receptor alpha	17	40309180	40357643
9.	SREBF2	Sterol regulatory element binding transcription factor 2	22	41833105	41907308
10.	CDK2	Cyclin dependent kinase 2	12	55966830	55972789
11.	MMP2	Matrix metallopeptidase 2	16	55478830	55506691
12.	ADORA2B	Adenosine A2b receptor	17	15927782	15975746
13.	GSK3A	Glycogen synthase kinase 3 alpha	19	42230186	42243330
14.	PLA2G1B	Phospholipase A2 group IB	12	120322115	120327779
15.	LDHA	Lactate dehydrogenase A	11	18394563	18408425
16.	CYP1B1	Cytochrome P450 family 1 subfamily B member 1	2	38067509	38076151
17.	MCL1	MCL1 apoptosis regulator, BCL2 family member	1	150574558	150579610
18.	G6PC	Glucose-6-phosphatase catalytic subunit	27	7903409	7906822
19.	CTSS	Cathepsin S	1	150730188	150765778
20.	DHFR	Dihydrofolate reductase	5	80626226	80654983
21.	NQO1	NAD(P)H quinone dehydrogenase 1	16	69709401	69726560
22.	DPP4	Dipeptidyl peptidase 4	2	161992245	162074215
23.	ICMT	Isoprenylcysteine carboxyl methyltransferase	1	6221193	6235964
24.	NCOR2	Nuclear receptor corepressor 2	12	124324415	124567612
25.	PDE2A	Phosphodiesterase 2A	11	72576141	72674422
26.	HDAC9	Histone deacetylase 9	7	18086825	19002416
27.	SLC8A1	Solute carrier family 8 member A1	2	40094523	40512452
28.	CYP	Cytochrome P450	1	24999844	25004308
29.	GRIA2	Glutamate ionotropic receptor AMPA type subunit 2	4	157220120	157370583
30.	EDNRB	Endothelin receptor type B	13	77895481	77975527
31.	PTPRCAP	Protein tyrosine phosphatase receptor type C associated protein	11	67435510	67437682
32.	CPA1	Carboxypeptidase A1	7	130380494	130388108
33.	SLC6A1	Solute carrier family 6 member 1	1	112911847	112956196
34.	CPT1A	Carnitine palmitoyltransferase 1A	11	68754620	68844410
35.	PTPN14	Protein tyrosine phosphatase non-receptor type 14	1	214348700	214551677

### Gene ontology enrichment analysis

**Figure 5:**
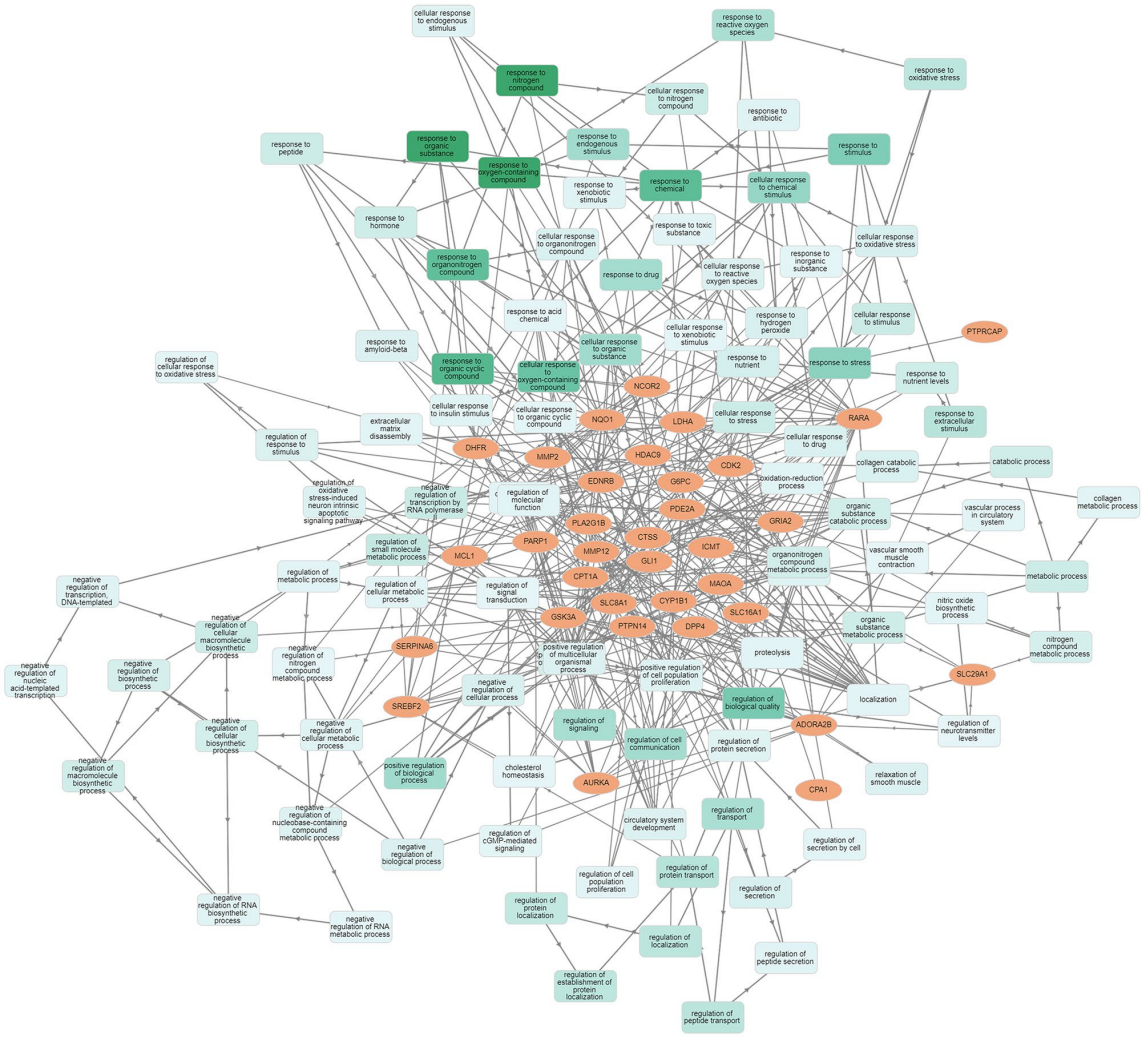
Representation of biological processes involved in the compound targeted thirty-five immune-responsive genes. The Orange color represents the immune targets and the green color represents the diverse biological processes.

The molecular features of the considerable HPV infected cervical cancer immune responsive genes interacting with compounds were further analyzed with the Metascape which revealed the involvement of these immune genes in different molecular functions and biological processes. The targeted immune-responsive genes and the corresponding proteins were attributed to be involved in the crucial biological regulation of signal transduction, cell population proliferation, cellular metabolic processes, proteolysis, cell communication, apoptosis, response to stress, catabolic process and oxidative process. These processes of biological regulation with the 35 immune genes were strongly evident in Fig. 5.

**Figure 6:**
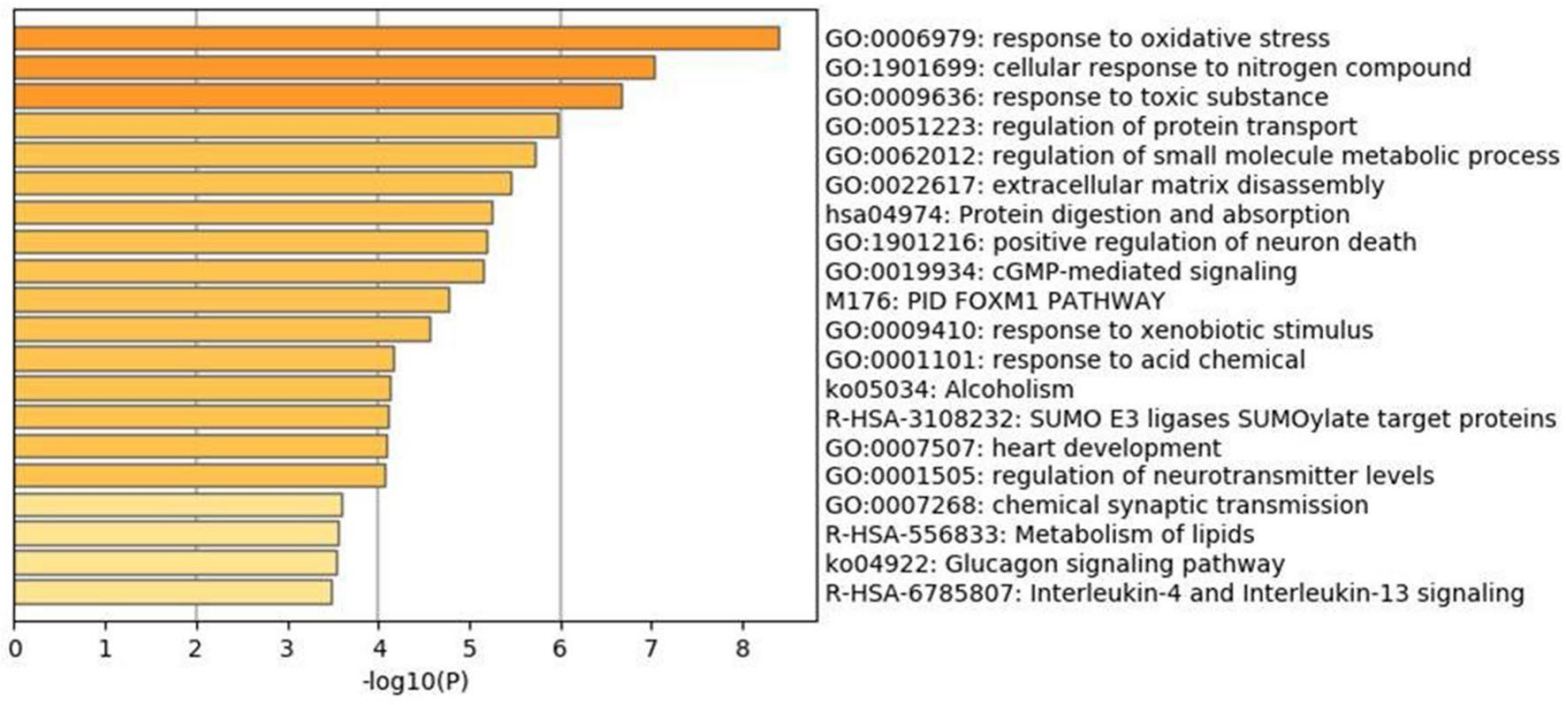
Histogram of Gene Ontology enrichment analysis corresponding to the *P-*value.

**Figure 7:**
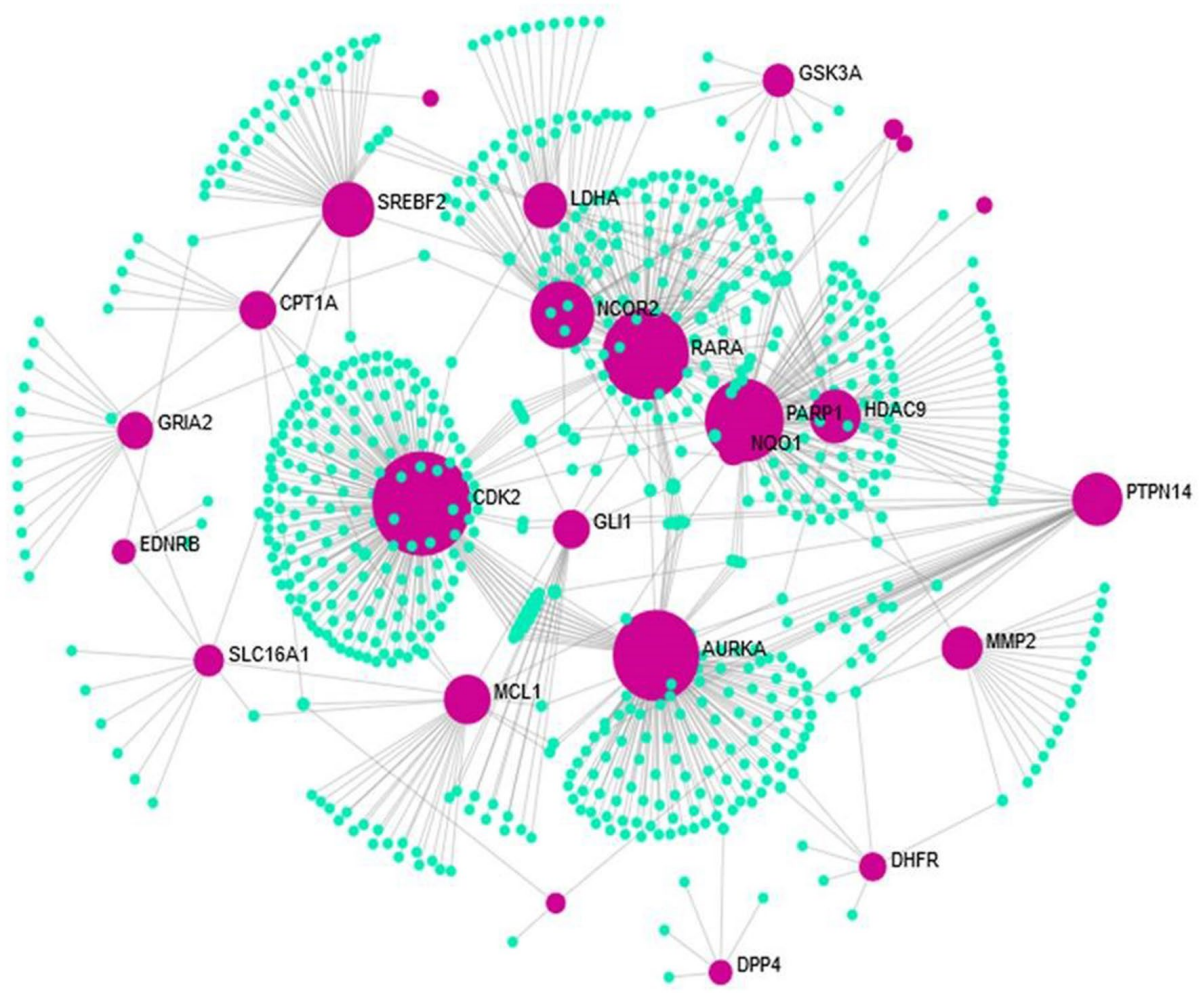
Tissue specific PPI obtained from Network Analyst. The pink colour in the image represents the significant immune responsive genes whereas the green color represents the interacting partners present in the human whole blood tissue.

The Molecular functions of the selected 35 immune genes targeted by compounds were represented in Supplementary Fig. S3 stating that the immune targets are responsible for catalytic activity and the histone deacetylases binding. Followed by the molecular function, the enrichment network analysis carried out with the Metascape database is represented in Supplementary Fig. S4 stating the involvement in various biological pathways. The histogram corresponding to the enriched pathways in relation to the identified 35 genes was represented in Fig. 6 and differentiated based on the cluster ID with saturated colors. Further, the 35 genes were analyzed for the tissue-specific PPI observed for the whole blood tissue type. These interactions are depicted in Fig. 7.

### Molecular interactome analysis

**Figure 8:**
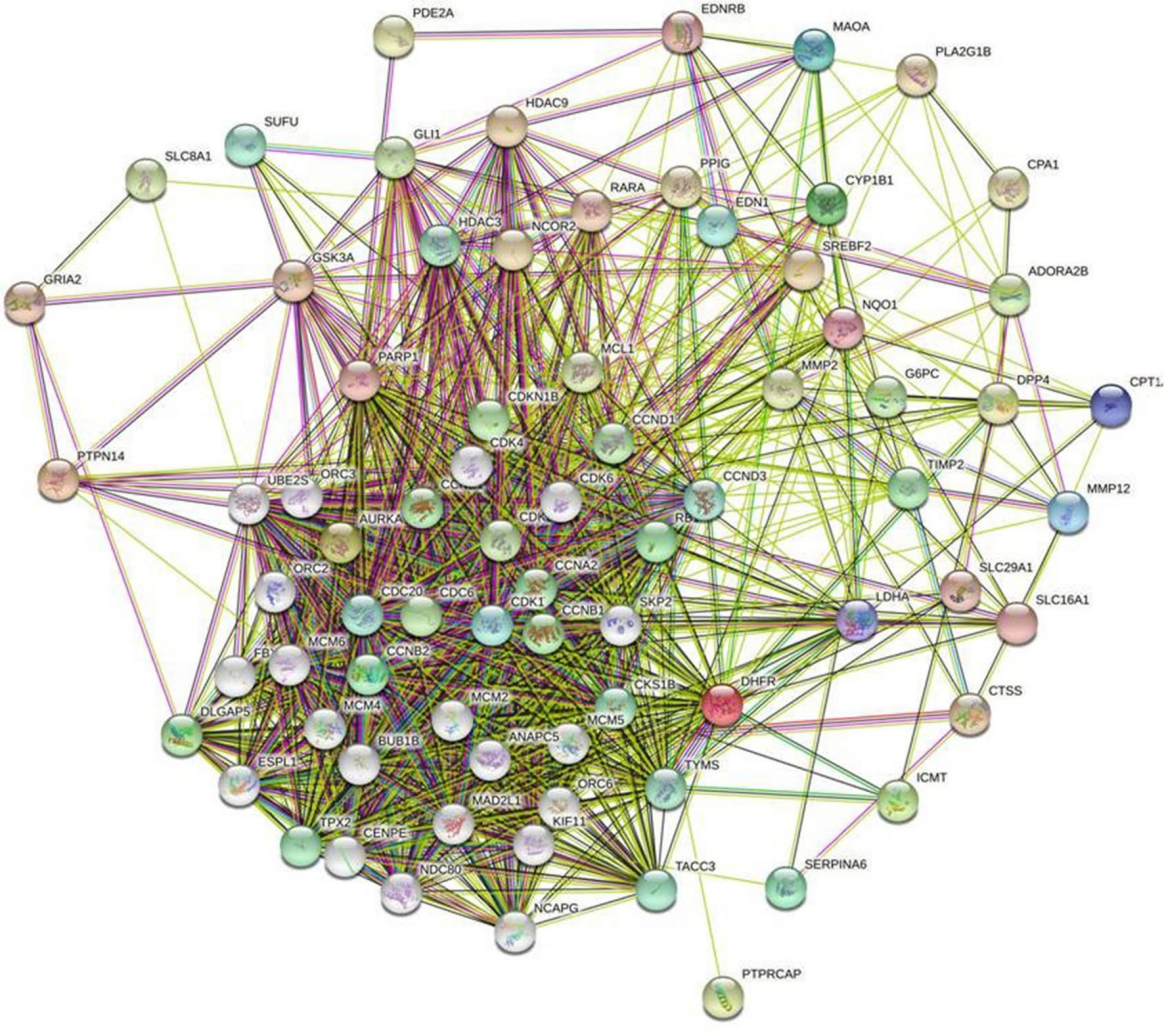
Immune targets and the molecular interactions of the cervical cancer immune target.

**Figure 9:**
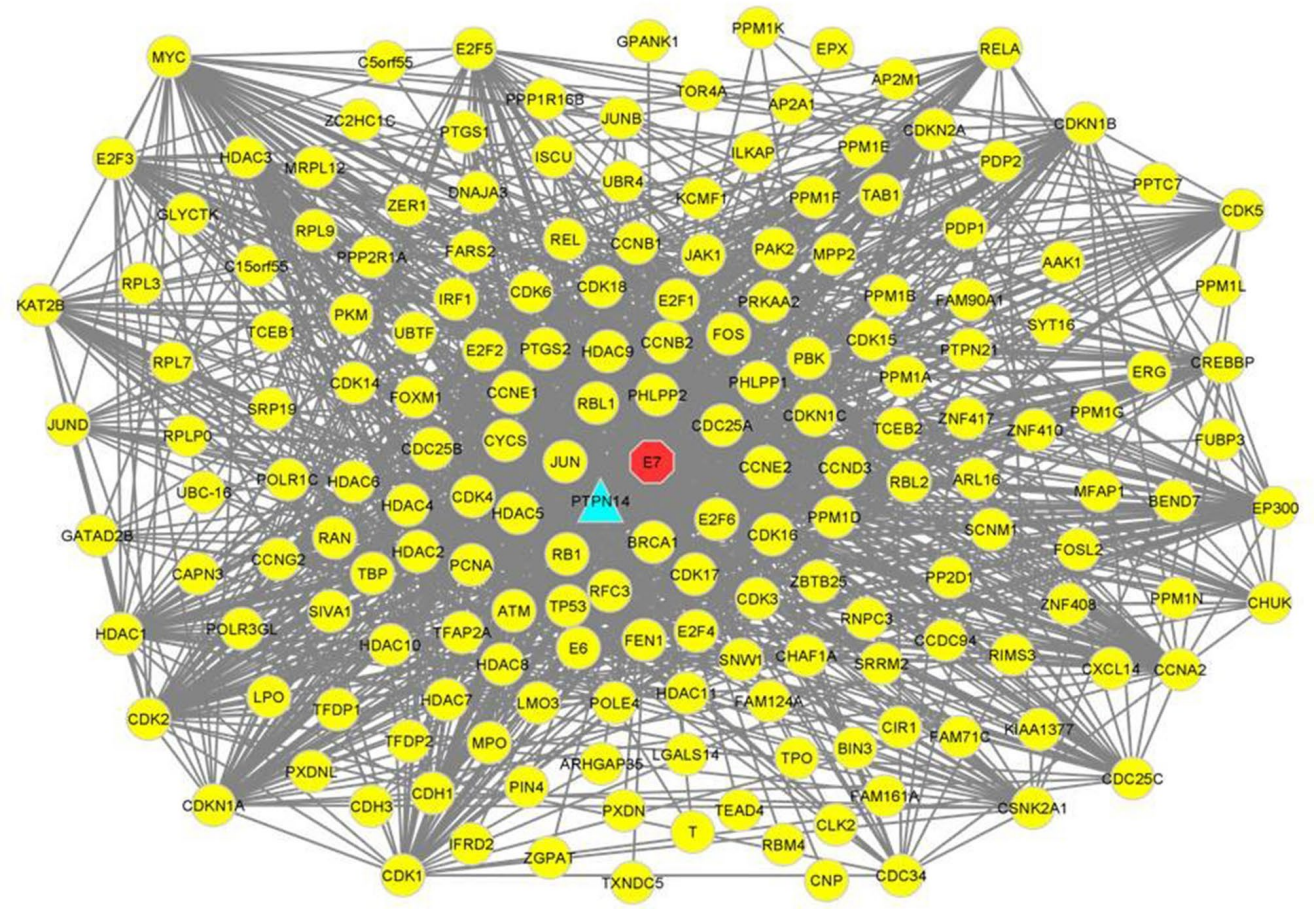
Molecular interactions between HPV E7 oncoprotein and the human proteins involved in cervical cancer.

A total of 35 genes including the cervical cancer immune genes retrieved from the compound network analysis and the literature reported which is differentially expressed between the healthy and the affected cases demonstrated the molecular cross-talks. The interactome possessess 75 nodes and 1101 edges represented in the Fig. 8. The average nodal degree of the immune responsive genes analyzed for the interactome is 29.4 in the closely connected immune proteins/genes. The enrichment score of the PPI for the immune responsive genes possesses *p*-value score of < 1.0e.16. These interactions also showed the complexity and functionalities of the cervical cancer responsive immune genes provided the potential targets for therapy against cancer. Additionally, the immune-responsive genes that interact with the HPV E7 obtained from the transcriptomic data were also identified for the molecular interaction between various human proteins which is represented in Fig. 9. This study clearly indicates that HPV E7 interacts with various cancer target proteins and represents those human proteins to be a potential target for drug discovery.

### Pharmacological features of the phytocompounds

The phytocompounds obtained from the literature reported for various biologically active plants have been calculated for their pharmacological properties such as the GPCR, Pi, Ki, Ncr, Ei, nVio which are represented in Table 4. The nVio and Ei has been considered to be significant with a threshold of above 0.5 feature score. Around 30 compounds are considered to be more efficacious that can be used further for the treatment of cervical cancer. The pharmacological features such as GPCR, Pi, Ki, Ncr, Ei, nVio influences the oral bioavailability, solubility and permeability of drug. These features were predicted through the experimentally validated computational approaches in accordance with the Rule of 5 (Ro5) drug discovery.

**Table 4:** Pharmacological features of the retrieved Phytocompounds.

S. no.	Compounds	GPCR	Ki	Ei	Pi	nVio
*M. indica*						
1.	Friedelin	0.02	− 0.39	0.21	0.02	1
2.	Humulene	− 0.14	− 0.93	0.31	− 0.67	1
3.	Elemene	− 0.36	− 1.02	0.30	− 0.38	1
4.	Epigallocatechin gallate	0.16	0.06	0.25	0.13	2
5.	Isomangiferin	0.04	0.05	0.47	− 0.08	2
6.	Linalool	− 0.73	− 1.26	0.07	− 0.94	0
7.	Β—Carotene	− 0.04	− 0.15	0.17	− 0.06	2
8.	B-sitosterol	0.14	− 0.51	0.51	0.07	1
9.	Octylgallate	− 0.15	− 0.25	0.05	− 0.24	0
10.	Linolenic acid	0.33	− 0.19	0.42	0.13	1
11.	Methyl gallate	− 0.89	− 0.89	− 0.36	− 1.03	0
12.	Ocimene	− 0.98	− 1.26	0.06	− 1.24	0
13.	Kaempferol	− 0.10	0.21	0.26	− 0.27	0
*N. sativa*						
14.	Thymoquinone	− 1.40	− 1.27	− 0.40	− 1.45	0
15.	Alpha hederin	− 0.91	− 1.89	− 0.90	− 0.56	3
16.	Nigellicine	− 0.15	− 0.18	0.20	− 0.57	0
17.	Nigellidine	0.07	0.29	0.19	− 0.32	0
18.	Thymohydroquinone	− 0.92	− 1.06	− 0.46	− 1.17	0
19.	Carvacrol	− 1.02	− 1.15	− 0.56	− 1.25	0
20.	Carvone	− 1.23	− 2.51	− 0.45	− 1.21	0
21.	Thymol	− 1.05	− 1.29	− 0.57	− 1.34	0
22.	Limonene	− 0.91	− 2.01	− 0.21	− 1.38	0
23.	4-Terpineol	− 0.56	− 1.68	0.06	− 0.92	0
24.	Alpha-pinene	− 0.48	− 1.50	− 0.34	− 0.85	0
25.	Tricyclene	− 0.81	− 1.36	− 058	− 0.94	0
26.	Camphene	− 1.02	− 1.85	− 0.82	− 1.40	0
27.	Sabinene	− 1.15	− 1.79	− 0.60	− 0.78	0
28.	1,8-Cineole	0.23	− 0.12	0.75	0.30	0
29.	Alpha—Terpinene	− 0.96	− 1.29	− 0.11	− 1.52	0
30.	Borneol	− 0.47	− 1.57	− 0.23	− 0.80	0
31.	Pinocarvone	− 0.77	− 2.06	− 0.38	− 0.67	0
32.	Cyclosativene	− 0.20	− 0.67	− 0.03	− 0.29	1
33.	Alpha-Longicyclene	-	-	-	-	-
34.	Alpha-Copaene	− 0.33	− 0.79	0.10	− 0.49	1
35.	Alpha—Longifolene	− 0.43	− 0.77	0.34	− 0.67	0
36.	Palmitic acid	0.02	− 0.33	0.18	− 0.04	1
37.	Octadecanoic acid	0.11	− 0.20	0.20	0.06	1
*Z. officinale*						
38.	6-Gingerol	0.16	− 0.33	0.38	0.15	0
39.	6-Shogaol	0.06	− 0.50	0.29	− 0.05	1
40.	6-paradol	− 0.01	− 0.47	0.18	− 0.09	0
41.	10-gingerol	0.18	− 0.24	0.32	0.21	1
42.	10-Shogaol	0.13	− 0.34	0.25	0.07	1
43.	6-dehydroshogaol	− 0.03	− 0.29	0.24	− 0.17	0
44.	Gingerenone	0.13	− 0.25	0.23	0.09	0
*C. grandis*						
45.	Naringin	0.11	− 0.24	0.24	0.09	0
46.	Nobiletin	− 0.13	0.09	0.11	− 0.22	0
47.	Tangeretin	− 0.12	0.06	0.11	− 0.20	0
48.	5-Demethyltangeretin	− 0.14	0.10	0.13	− 0.27	0
49.	Sinensetin	− 0.08	0.14	0.10	− 0.20	0
50.	Naringenin	0.03	− 0.26	0.21	− 0.12	0
51.	Hesperidin	− 0.01	− 0.36	0.06	− 0.00	3
52.	Methoxylated	− 0.77	− 0.99	− 0.41	− 0.86	0
*Z. jujube*						
53.	Ursolic acid	0.28	− 0.50	0.69	0.23	1
54.	Oleanolic acid	0.28	− 0.40	0.65	0.15	1
55.	Betulinic acid	0.31	− 0.50	0.55	0.14	1
*Z. Mauritiana*						
56.	Glaucine	0.37	− 0.09	0.13	− 0.09	0
*C. cassia*						
57.	Cinnamaldehyde	− 1.09	− 1.24	− 0.46	− 0.79	0
58.	Cinnamic acid	− 0.74	− 1.14	− 0.30	− 0.98	0
59.	Cinnamyl acetate	− 0.71	− 1.04	− 0.23	− 0.94	0
60.	-Thujene	− 0.96	− 1.79	− 0.58	− 1.02	0
61.	-Terpineol	− 0.51	− 1.45	0.14	− 0.78	0
62.	-Cubebene	− 0.50	− 0.75	− 0.24	− 0.35	1
63.	Eugenol	− 0.86	− 1.14	− 0.41	− 1.29	0
64.	-Caryophyllene	− 0.34	− 0.78	0.19	− 0.60	1
65.	Terpinolene	− 0.88	− 1.61	− 0.26	− 1.74	0
66.	E-Nerolidol	− 0.17	− 0.64	0.39	− 0.43	1
67.	L-Borneol	− 0.47	− 1.57	− 0.23	− 0.80	0
68.	Caryophyllene Oxide	− 0.08	− 0.86	0.57	0.00	0
69.	Coumarin	− 1.44	− 1.57	− 0.58	− 1.43	0
70.	Myrcene	− 1.11	− 1.51	− 0.07	− 1.31	0
71.	Alpha-Phellandrene	− 1.00	− 1.40	− 0.15	− 1.38	0
72.	Terpinolene	− 0.88	− 1.61	− 0.26	− 1.74	0
73.	Isoborneol	− 0.47	− 1.57	− 0.23	− 0.80	0
74.	Geraniol	− 0.60	− 1.32	0.28	− 1.03	0
75.	Safrole	− 0.84	− 1.27	− 0.49	− 1.24	0
76.	Phenylacetaldehyde	− 2.16	− 2.39	− 1.57	− 1.82	0
77.	Vanillin	− 1.20	− 1.13	− 0.64	− 1.65	0
78.	Salicylaldehyde	− 2.53	− 2.38	− 1.83	− 2.76	0
79.	Acetophenone	− 2.46	− 2.82	− 1.97	− 2.63	0
80.	Anisaldehyde	− 1.44	− 1.40	− 0.89	− 1.79	0
81.	Beta-Bisobolol	− 0.20	− 0.88	0.36	− 0.52	0
82.	Alpha-Muurolol	− 0.20	− 0.88	0.36	− 0.52	0
83.	Patchoulene	− 0.52	− 1.22	− 0.13	− 0.59	0
84.	Guaicol	− 2.29	− 2.30	− 1.75	− 2.62	0
85.	Methyl alaninate	− 3.46	− 3.65	− 3.26	− 3.02	0
86.	Undecanoic acid	− 0.36	− 0.88	− 0.01	− 0.46	0
87.	Decanoic acid	− 0.46	− 1.03	− 0.07	− 0.56	0

## Discussion

Cancer has been represented to be the most widespread cause of mortality worldwide which leads to millions of deaths every year. This has been caused by various infections by different microorganisms among which viral infections catch hold of 20 percent importance and represent a significant function in the development of malignant and benign tumors^46^. These viruses contain various genes and proteins which possess oncogenic properties facilitating the various stages of carcinogenesis. One of the oncogenic viruses is the HPV which is involved in the infection through sexual transmission and causes cervical cancer^47^. Reports have witnessed stating that the oncoproteins of HPV interact with known and unknown cellular factors which are highly responsible for the development of cancerous lesions^42,48^.

Numerous drugs and vaccines are reported for the treatment of cervical cancer but the ineffectiveness of the anti-HPV drugs to medicate the harmful infection provoked us to identify the novel compounds that are effective in the control and treatment of the cancerous growth in humans. Alongside, the use of traditional medicines is known for decades in the treatment of various diseases in this world through the ancient medical practices^49^. Even though, the chemical composition of the medicinal plants has been obtained for the flawless drug development, this aspect is not confident enough due to the higher insolence of chemical entities and the functional aspects of the drug statute^33,50^. Researchers have reported that traditional medicines were proven to be effective to treat the infections and diseases caused by viruses like HIV, measles, hepatitis, coxsackievirus and HPV^50^. These observations on the viral oncoproteins and the role of plant compounds provided more insights into the understanding of the interaction with the human physiological system through the control of molecular cross-talks between the key elements in the immunological aspects. Further, the exact mechanism of the immune responsive targets and the impression of herbal medicines to treat viral infections is inadequate still^35^. With this as pilot information, we presented the immuno-transcriptomics and systems pharmacology strategies to unravel the immune targets of HPV and the associated signaling pathways along with the pharmacological roles of *M. indica, N. sativa, Z officinale, C. grandis, Z. jujube, Z. mauritiana* and *C. cassia* derived bioactive compounds for the treatment of HPV related cancerous growth at molecular level. Additionally, the information on the bioactive molecules derived from the natural plants for the treatment of viral disease provides vital information on therapeutics.

Our study is mainly focused on the identification and understanding of the pattern related to the gene expression of the host which is responsible for cervical cancer. Our investigation lies with the performance of the immuno-transcriptomics profiling between the infected and healthy controls with the transcriptomics dataset available in the public databases. These datasets were further processed with the Network Analyst 3.0 which helps in the retrieval of heatmap representing the intensities of microarray in cervical cancer immune genes. Based on this analysis, a total of 384 immune responsive genes between the healthy and infected patients were obtained with the intensity values. Further, these genes were selected for the successive analysis of the PPI. On the other hand, PubChem and other omics databases help in identifying 87 phytocompounds that are responsible for treating cervical cancer. Among the 87 phytocompounds, 79 compounds interacted with the 35 differentially expressed cervical cancer associated immune genes through drug targeting. The compounds that interact with the immune responsive genes were represented in Table 2 which shows that only 35 immune genes are involved in the process of drug targeting among the 87 phytocompounds. Remarkably, the predicted immune genes which are involved in the different biological activities against cervical cancer has been identified and some of the genes obtained from network analyst are not reported till date which exhibits the capability of SwissTargetPrediction and the gene ontology enrichment evaluation methods. Further, the ORA functional enrichment of the identified genes was demarcated with the help of Metascape. The analysis of the compounds that target the human genes which are expressed differentially highlights the role of 35 genes among the identified 384 genes and the literature reported receptors. The immune-responsive genes PTPN14, CDK2, HDAC9, MMP2, AURKA, PARP1 and GRIA depicts their role in most of the diseases targeting humans among the highlighted 35 components. The genes CDK2, HDAC9 and PTPN14 interact commonly with the phytocompounds that target the immune responsive and also with the HPV E7 oncoprotein which is strongly evident in Fig. 9. For instance, PTPN14 (Protein Tyrosine Phosphatase Non-Receptor Type 14) is the potential tumor suppressor which owns its involvement in the linkage to the control of Hippo and the Wnt/beta-catenin signaling pathways. Herein, we understood that the cross-talk between HPV E7 and PTPN14 might be the important reason for immune-pathological expression of cervical cancer^51^. The genes which have been commonly targeted by various phytocompounds are significantly playing a noteworthy role in different viral infections and are also responsible for other cancers/diseases.

The correlation between the host immune response to catalytic activity and the histone deacetylases binding is identified through the classification of molecular functions. The obtained results revealed that, the immune response between infections and cervical cancer is highly significant at the biological level. As per our earlier discussion, the role of PTPN14, CDK2 and HDAC9 in tumor suppression and the interaction with HPV E7 oncoprotein is observed stating that these targets can be potential druggable targets for cervical cancer. Additionally, functional enrichment and the gene ontology revealed its strong relation to apoptosis and other pathways in cancer. The molecular interactome analysis between the viral oncoprotein and the components of human immune response demonstrates the critical capability of viral replication to dodge the immunological responses. The compound target network analysis revealed that the retrieved 79 among 87 compounds strongly bind with the identified 35 compounds representing its ability to inhibit the progression of any diseases. Further, the cytoscape analysis revealed the interaction between the phytocompounds and the human immune genes which is the essential identification of this study. This study unveils the diversified mechanism of the compounds and the plausible mode of action towards assorted targets involved in cancer. These inferences help us to put forth the curative effects and promote the use of traditional medicines that leads to make novel avenues in the field of drug discovery and development. Further, it impacts the people’s lives by providing the drugs at low cost.

## Conclusion

The results from our study reveals that the Indian traditional phytocompounds including a variety of medicinal values exhibits the expanded immunological stimulants to treat cervical cancer caused by the infection of HPV E7. The research on the traditional Indian medical plants notably on *M. indica, N. sativa, Z. officinale, C. grandis, Z.jujube, Z. mauritiana* and *C. cassia* is not explored completely. Radically, our result on the phytocompounds with the pharmacological properties represents the interaction with the human immune responsive genes along with their role in various biological processes and function. These studies overlay the groundwork for the aperture of advanced biological research on different types of cancer with the combination of traditional medicines. This study identified the several crucial aspects of the host immune response towards the infection of HPV E7 oncoprotein. The identified immune responsive genes and the corresponding signaling pathways help in unraveling the pathogenesis of cervical cancer. Also, these analyses help in the characterization of the immuno-pathology of the HPV E7 infection. Our study is mainly focused on the conjecturing the use of phytocompounds in combination with other components may provide synergistic effects which may lead to further development of new anti-HPV or anti-cancer drugs. Additionally, the molecular interactome analysis reveals that thirty-eight immune responsive genes can be considered the effective druggable targets for the treatment of various cancer and other diseases. On the whole, this comprehensive study behaves as the stage to improve the understanding of the immunological behavior of the HPV E7 oncoprotein which also provides the widespread knowledge in executing the intrusion approach.

## Supplementary Information

Supplementary Figures.

Supplementary Table S1.

Supplementary Table S2.

## Data Availability

The datasets generated and/or analyzed during the current study are not publicly available since the continuation of the work has not been published but are available from the corresponding author on reasonable request.
