# Supplementary material for: Unraveling the multi-targeted curative potential of bioactive molecules against cervical cancer through integrated omics and systems pharmacology approach
Source: Sci Rep. 2022 Aug 21;12:14245. doi: 10.1038/s41598-022-18358-7 (PMC9393168; doi:10.1038/s41598-022-18358-7)
Supplement: Supplementary file 1 — Supplementary Figures. [file 41598_2022_18358_MOESM1_ESM.docx]

**
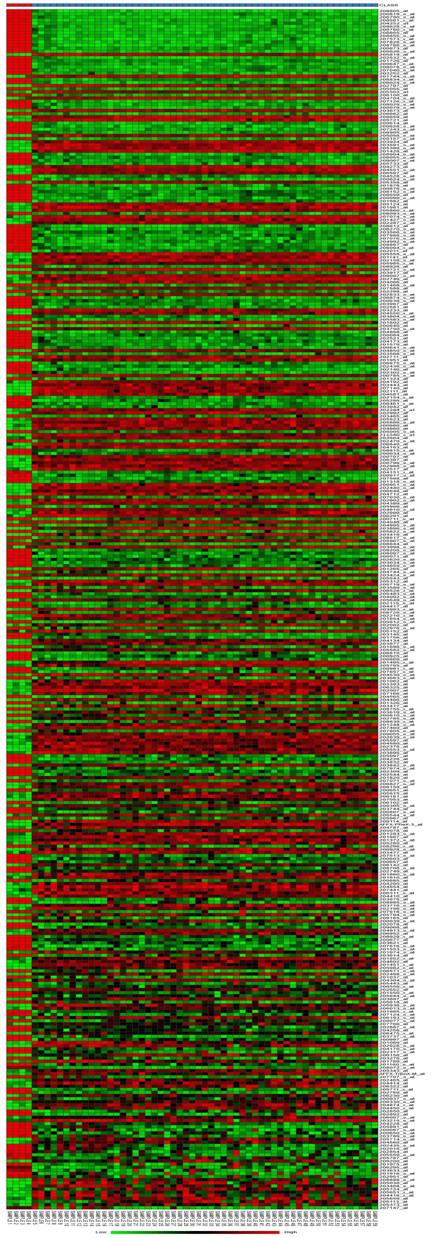
**

**Supplementary Figure S1: Heat map of the differentially expressed genes**

**
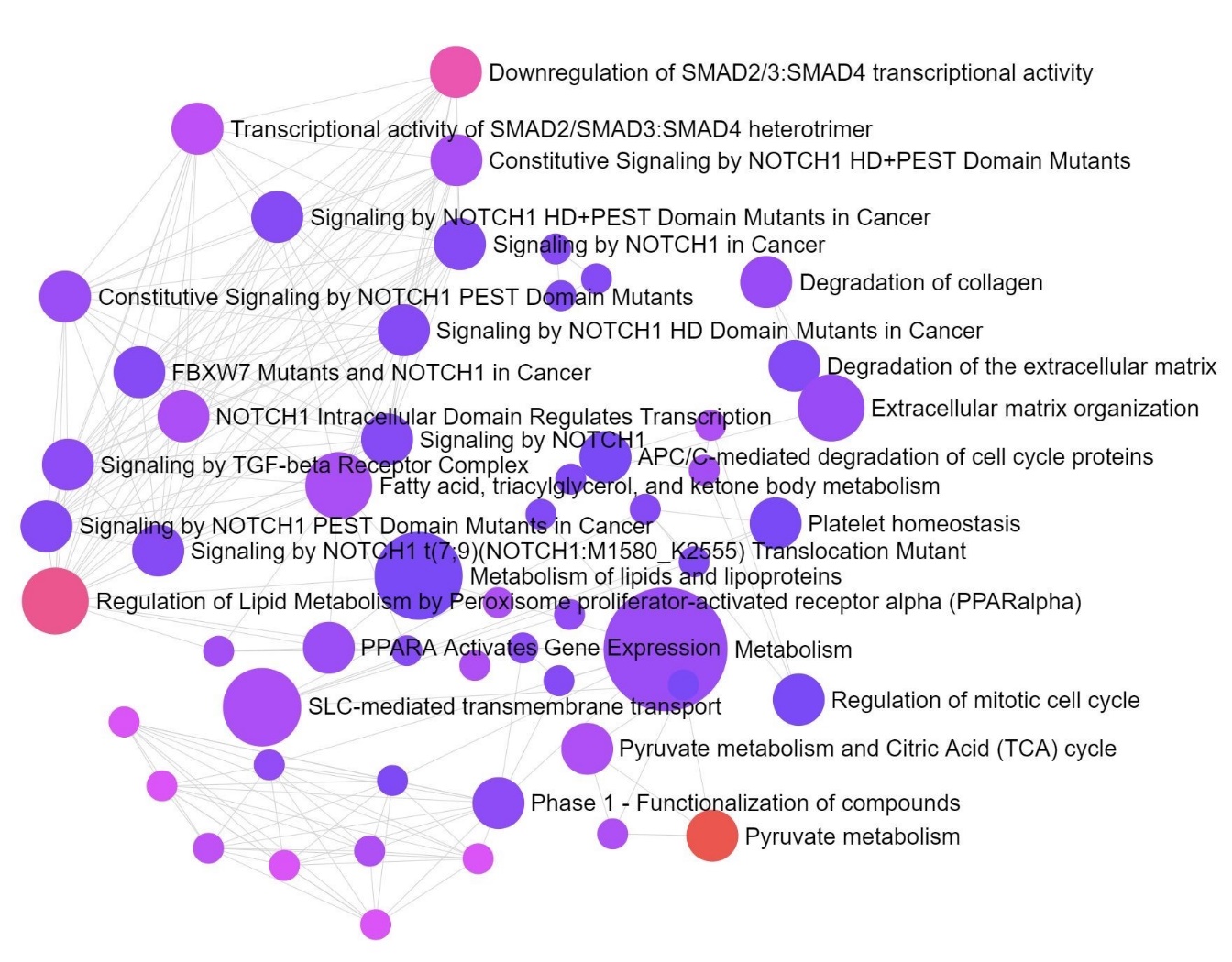
**

**Supplementary Figure S2: Biological function of the significant genes which has been observed through network analyst**

**
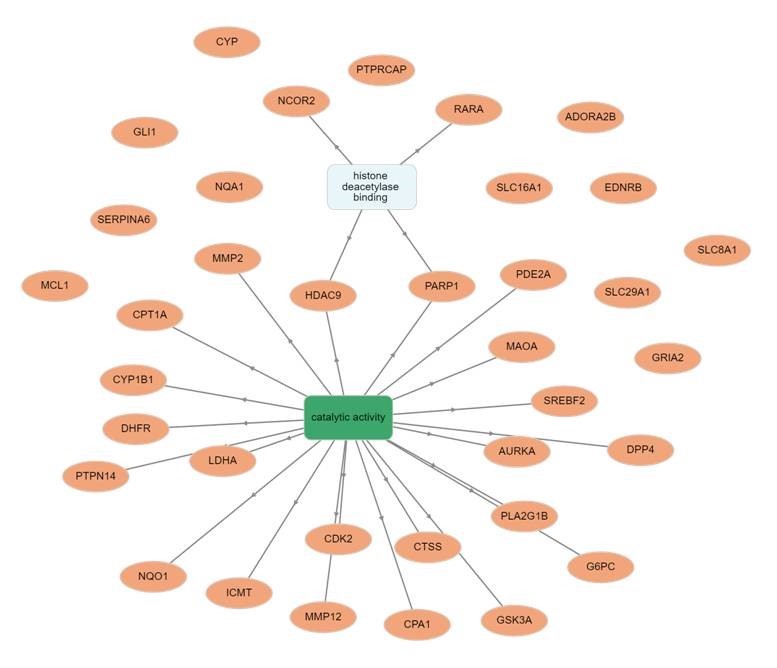
**

**Supplementary Figure S3: Representation of Molecular function involved in the compound targeted thirty five immune responsive genes in which orange color represents the immune targets and the green color represents the molecular functions.**

**
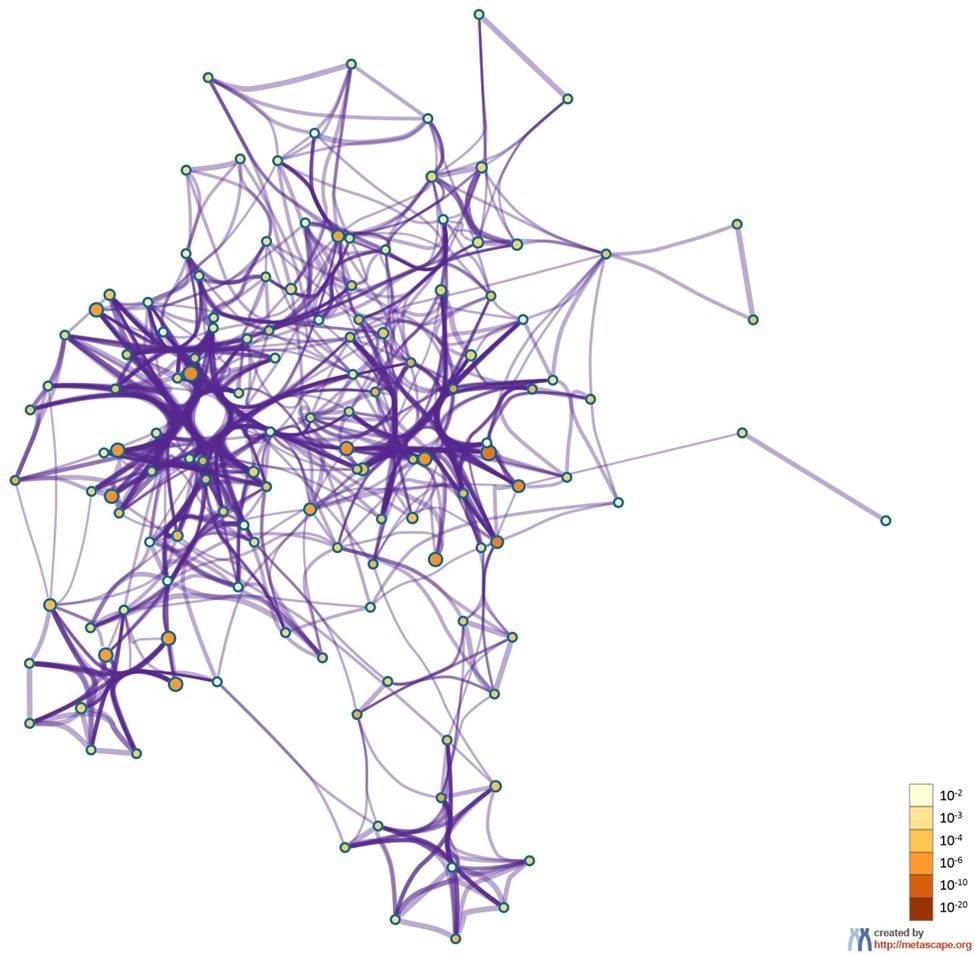
**

**Supplementary Figure S4: Enrichment network analysis representation colored by *P*-value and the colored spheres represent the pathways which is enclosed y more genes tend to have momentous *P*-value**
