# Supplementary material for: Unraveling the multi-targeted curative potential of bioactive molecules against cervical cancer through integrated omics and systems pharmacology approach
Source: Sci Rep. 2022 Aug 21;12:14245. doi: 10.1038/s41598-022-18358-7 (PMC9393168; doi:10.1038/s41598-022-18358-7)
Supplement: Supplementary file 2 — Supplementary Table S1. [file 41598_2022_18358_MOESM2_ESM.docx]

**Supplementary Table S1: Gene names obtained with the BioGPS database.**

| **No** | **Probe Set ID** | **Gene Symbol** | **Gene Names** | **No** | **Probe Set ID** | **Gene Symbol** | **Gene Names** |
| --- | --- | --- | --- | --- | --- | --- | --- |
|  | 208805_at | - | - |  | 200819_s_at | RPS15 | ribosomal protein S15 |
|  | 206790_s_at | NDUFB1 | NADH:ubiquinoneoxidoreductase subunit B1) |  | 208581_x_at | MT1X | metallothionein 1X |
|  | 204252_at | CDK2 | cyclin dependent kinase 2 |  | 208628_s_at | YBX1 | Y-box binding protein 1 |
|  | 208780_x_at | VAPA | VAMP associated protein A |  | 208865_at | CSNK1A1 | casein kinase 1 alpha 1 |
|  | 208855_s_at | STK24 | Serine/threonine kinase 24 |  | 207573_x_at | ATP5MG | ATP synthase membrane subunit g |
|  | 207828_s_at | CENPF | centromere protein F |  | 208786_s_at | MAP1LC3B | microtubule associated protein 1 light chain 3 beta |
|  | 200673_at | LAPTM4A | lysosomal protein transmembrane 4 alpha |  | 200626_s_at | MATR3 | matrin 3 |
|  | 205819_at | MARCO | macrophage receptor with collagenous structure |  | 202532_s_at | DHFR | dihydrofolatereductase |
|  | 201726_at | ELAVL1 | ELAV like RNA binding protein 1 |  | 200647_x_at | EIF3C | eukaryotic translation initiation factor 3 subunit C |
|  | 208078_s_at | SIK1 | salt inducible kinase 1 |  | 207040_s_at | ST13 | ST13 Hsp70 interacting protein |
|  | 202252_at | RAB13 | RAB13, member RAS oncogene family |  | 201744_s_at | LUM | lumican |
|  | 208834_x_at | RPL23A | ribosomal protein L23a |  | 209024_s_at | SYNCRIP | synaptotagmin binding cytoplasmic RNA interacting protein |
|  | 202707_at | UMPS | uridine monophosphate synthetase |  | 205055_at | ITGAE | integrin subunit alpha E |
|  | 205503_at | PTPN14 | protein tyrosine phosphatase non-receptor type 14 |  | 206100_at | CPM | carboxypeptidase M |
|  | 204704_s_at | ALDOB | aldolase, fructose-bisphosphate B |  | 207126_x_at | UGT1A10 | UDP glucuronosyltransferase family 1 member A10 |
|  | 208029_s_at | LAPTM4B | lysosomal protein transmembrane 4 beta |  | 200079_s_at | KARS1 | lysyl-tRNAsynthetase 1 |
|  | 203673_at | TG | thyroglobulin |  | 206662_at | GLRX | glutaredoxin |
|  | 206659_at | - | - |  | 205721_at | GFRA2 | GDNF family receptor alpha 2 |
|  | 200614_at | CLTC | clathrin heavy chain |  | 200628_s_at | WARS1 | tryptophanyl-tRNAsynthetase 1 |
|  | 207243_s_at | CALM3 | calmodulin 3 |  | 208905_at | CYCS | cytochrome c, somatic |
|  | 205056_s_at | GPR162 | G protein-coupled receptor 162 |  | 205157_s_at | KRT17 | keratin 17 |
|  | 203924_at | GSTA1 | glutathione S-transferase alpha 1 |  | 203591_s_at | CSF3R | colony stimulating factor 3 receptor |
|  | 205390_s_at | ANK1 | ankyrin 1 |  | 201428_at | CLDN4 | claudin 4 |
|  | 208904_s_at | RPS28 | ribosomal protein S28 |  | 206055_s_at | SNRPA1 | small nuclear ribonucleoprotein polypeptide A' |
|  | 209007_s_at | RSRP1 | arginine and serine rich protein 1 |  | 205733_at | BLM | BLM RecQ like helicase |
|  | 204273_at | EDNRB | endothelin receptor type B |  | 204551_s_at | AHSG | alpha 2-HS glycoprotein |
|  | 206597_at | NRL | neural retina leucine zipper |  | 204528_s_at | NAP1L1 | nucleosome assembly protein 1 like 1 |
|  | 200624_s_at | SNHG4 | small nucleolar RNA host gene 4 |  | 205358_at | GRIA2 | glutamate ionotropic receptor AMPA type subunit 2 |
|  | 201878_at | ARIH1 | ariadne RBR E3 ubiquitin protein ligase 1 |  | 200876_s_at | PSMB1 | proteasome 20S subunit beta 1 |
|  | 208152_s_at | DDX21 | DExD-box helicase 21 |  | 208659_at | CLIC1 | chloride intracellular channel 1 |
|  | 200099_s_at | RPS3A | ribosomal protein S3A |  | 201682_at | PMPCB | peptidase, mitochondrial processing subunit beta |
|  | 205124_at | BORCS8 | BLOC-1 related complex subunit 8 |  | 201981_at | PAPPA | pappalysin 1 |
|  | 205860_x_at | FOLH1B | folate hydrolase 1B |  | 208093_s_at | NDEL1 | nudE neurodevelopment protein 1 like 1 |
|  | 207074_s_at | SLC18A1 | solute carrier family 18 member A1 |  | 201427_s_at | SELENOP | selenoprotein P |
|  | 202287_s_at | TACSTD2 | tumor associated calcium signal transducer 2 |  | 208612_at | PDIA3 | protein disulfide isomerase family A member 3 |
|  | 208270_s_at | RNPEP | arginylaminopeptidase |  | 203566_s_at | AGL | amylo-alpha-1, 6-glucosidase, 4-alpha-glucanotransferase |
|  | 207988_s_at | ARPC2 | actin related protein 2/3 complex subunit 2 |  | 207076_s_at | ASS1 | argininosuccinate synthase 1 |
|  | 204992_s_at | PFN2 | profilin 2 |  | 208887_at | EIF3G | eukaryotic translation initiation factor 3 subunit G |
|  | 206094_x_at | UGT1A5 | UDP glucuronosyltransferase family 1 member A5 |  | 202011_at | TJP1 | tight junction protein 1 |
|  | 205555_s_at | MSX2 | mshhomeobox 2 |  | 201141_at | GPNMB | glycoprotein nmb |
|  | 202158_s_at | CELF2 | CUGBP Elav-like family member 2 |  | 205985_x_at | CLCNKB | chloride voltage-gated channel Kb |
|  | 208926_at | NEU1 | neuraminidase 1 |  | 200721_s_at | ACTR1A | actin related protein 1A |
|  | 203917_at | CXADR | CXADR Ig-like cell adhesion molecule |  | 209097_s_at | JAG1 | jagged canonical Notch ligand 1 |
|  | 202746_at | ITM2A | integral membrane protein 2A |  | 204086_at | PRAME | PRAME nuclear receptor transcriptional regulator |
|  | 201468_s_at | NQO1 | NAD(P)H quinone dehydrogenase 1 |  | 207588_at | - | - |
|  | 202298_at | NDUFA1 | NADH:ubiquinoneoxidoreductase subunit A1 |  | 202833_s_at | SERPINA1 | serpin family A member 1 |
|  | 206874_s_at | SLK | STE20 like kinase |  | 200639_s_at | YWHAZ | tyrosine 3-monooxygenase/tryptophan 5-monooxygenase activation protein zeta |
|  | 203967_at | CDC6 | cell division cycle 6 |  | 202581_at | HSPA1B | heat shock protein family A (Hsp70) member 1B |
|  | 203233_at | IL4R | interleukin 4 receptor |  | 204550_x_at | GSTM1 | glutathione S-transferase mu 1 |
|  | 203804_s_at | LUC7L3 | LUC7 like 3 pre-mRNA splicing factor |  | 205583_s_at | ALG13 | ALG13 UDP-N-acetylglucosaminyltransferase subunit |
|  | 201802_at | SLC29A1 | solute carrier family 29 member 1 (Augustine blood group) |  | 200640_at | YWHAZ | tyrosine 3-monooxygenase/tryptophan 5-monooxygenase activation protein zeta |
|  | 203750_s_at | RARA | retinoic acid receptor alpha |  | 204868_at | MRPL58 | mitochondrial ribosomal protein L58 |
|  | 200084_at | C11orf58 | chromosome 11 open reading frame 58 |  | 202521_at | CTCF | CCCTC-binding factor |
|  | 204173_at | MYL6B | myosin light chain 6B |  | 201570_at | SAMM50 | SAMM50 sorting and assembly machinery component |
|  | 200641_s_at | YWHAZ | tyrosine 3-monooxygenase/tryptophan 5-monooxygenase activation protein zeta |  | 204850_s_at | DCX | doublecortin |
|  | 203568_s_at | TRIM38 | tripartite motif containing 38 |  | 202711_at | EFNB1 | ephrin B1 |
|  | 201951_at | ALCAM | activated leukocyte cell adhesion molecule |  | 202146_at | IFRD1 | interferon related developmental regulator 1 |
|  | 202436_s_at | CYP1B1 | cytochrome P450 family 1 subfamily B member 1 |  | 200785_s_at | LRP1 | LDL receptor related protein 1 |
|  | 202302_s_at | RSRC2 | arginine and serine rich coiled-coil 2 |  | 204192_at | CD37 | CD37 molecule |
|  | 201324_at | EMP1 | Epithelial membrane protein 1 |  | 207140_at | ALPI | alkaline phosphatase, intestinal |
|  | 203443_at | EML3 | EMAP like 3 |  | 204581_at | CD22 | CD22 molecule |
|  | 202111_at | SLC4A2 | solute carrier family 4member 2 |  | 205286_at | TFAP2C | transcription factor AP-2 gamma |
|  | 202154_x_at | TUBB3 | tubulin beta 3 class III |  | 203042_at | LAMP2 | lysosomal associated membrane protein 2 |
|  | 206461_x_at | MT1H | metallothionein 1H |  | 202992_at | C7 | complement C7 |
|  | 202284_s_at | CDKN1A | cyclin dependent kinase inhibitor 1A |  | 205523_at | HAPLN1 | hyaluronan and proteoglycan link protein 1 |
|  | 203465_at | MRPL19 | mitochondrial ribosomal protein L19 |  | 200986_at | SERPING1 | serpin family G member 1 |
|  | 205686_s_at | CD86 | CD86 molecule |  | 205005_s_at | NMT2 | N-myristoyltransferase 2 |
|  | 204960_at | PTPRCAP | protein tyrosine phosphatase receptor type C associated protein |  | 202664_at | WIPF1 | WAS/WASL interacting protein family member 1 |
|  | 211540_s_at | RB1 | RB transcriptional corepressor 1 |  | 206840_at | AFM | afamin |
|  | 202470_s_at | CPSF6 | cleavage and polyadenylation specific factor 6 |  | 208815_x_at | HSPA4 | heat shock protein family A (Hsp70) member 4 |
|  | 204163_at | EMILIN1 | elastin microfibrilinterfacer 1 |  | 200707_s_at |  |  |
|  | 200034_s_at | RPL6 | ribosomal protein L6 |  | 200796_s_at | MCL1 | MCL1 apoptosis regulator, BCL2 family member |
|  | 206387_at | CDX2 | caudal type homeobox 2 |  | 202517_at | CRMP1 | collapsin response mediator protein 1 |
|  | 202988_s_at | RGS1 | regulator of G protein signaling 1 |  | 200917_s_at | SRPRA | SRP receptor subunit alpha |
|  | 204151_x_at | - | - |  | 201318_s_at | MYL12B | myosin light chain 12B |
|  | 207508_at | ATP5MC3 | ATP synthase membrane subunit c locus 3 |  | 202440_s_at | DENND2B | DENN domain containing 2B |
|  | 200951_s_at | CCND2 | cyclin D2 |  | 204712_at | WIF1 | WNT inhibitory factor 1 |
|  | 206646_at | GLI1 | GLI family zinc finger 1 |  | 202902_s_at | CTSS | cathepsin S |
|  | 207030_s_at | CSRP2 | cysteine and glycine rich protein 2 |  | 205909_at | POLE2 | DNA polymerase epsilon 2, accessory subunit |
|  | 204389_at | MAOA | monoamine oxidase A |  | 202989_at | RGS1 | regulator of G protein signaling 1 |
|  | 204910_s_at | TRIM3 | tripartite motif containing 3 |  | 200711_s_at | SKP1 | S-phase kinase associated protein 1 |
|  | 206291_at | NTS | neurotensin |  | 204905_s_at | EEF1E1 | eukaryotic translation elongation factor 1 epsilon 1 |
|  | 204046_at | PLCB2 | phospholipase C beta 2 |  | 205822_s_at | HMGCS1 | 3-hydroxy-3-methylglutaryl-CoA synthase 1 |
|  | 203896_s_at | PLCB4 | phospholipase C beta 4 |  | 208617_s_at | PTP4A2 | (protein tyrosine phosphatase 4A2 |
|  | 203416_at | CD53 | CD53 molecule |  | RARA | PARP1 | poly(ADP-ribose) polymerase 1 |
|  | 205907_s_at | OMD | osteomodulin |  | 209205_s_at | LMO4 | LIM domain only 4 |
|  | 203998_s_at | SYT1 | synaptotagmin 1 |  | 200071_at | SMNDC1 | survival motor neuron domain containing 1 |
|  | 208097_s_at | TMX1 | thioredoxin related transmembrane protein 1 |  | 203034_s_at | RPL27A | ribosomal protein L27a |
|  | 202620_s_at | PLOD2 | procollagen-lysine,2-oxoglutarate 5-dioxygenase 2 |  | 201265_at | - | - |
|  | 204659_s_at | GFER | growth factor, augmenter of liver regeneration |  | 203424_s_at | IGFBP5 | insulin like growth factor binding protein 5 |
|  | 204744_s_at | IARS1 | isoleucyl-tRNAsynthetase 1 |  | 205312_at | SPI1 | Spi-1 proto-oncogene |
|  | 205543_at | HSPA4L | heat shock protein family A (Hsp70) member 4 like |  | 203589_s_at | TFDP2 | transcription factor Dp-2 |
|  | 205719_s_at | PAH | phenylalanine hydroxylase |  | 205483_s_at | ISG15 | ISG15 ubiquitin like modifier |
|  | 208528_x_at | SSX7 | SSX family member 7 |  | 205649_s_at | FGA | fibrinogen alpha chain |
|  | 204092_s_at | AURKA | aurora kinase A |  | 204417_at | GALC | galactosylceramidase |
|  | 202115_s_at | NOC2L | NOC2 like nucleolar associated transcriptional repressor |  | 208720_s_at | RBM39 | RNA binding motif protein 39 |
|  | 203993_x_at | CFAP410 | cilia and flagella associated protein 410 |  | 201654_s_at | HSPG2 | heparan sulfate proteoglycan 2 |
|  | 202210_x_at | GSK3A | glycogen synthase kinase 3 alpha |  | 202002_at | ACAA2 | acetyl-CoA acyltransferase 2 |
|  | 205812_s_at | TMED9 | transmembrane p24 trafficking protein 9 |  | 205152_at | SLC6A1 | solute carrier family 6 member 1 |
|  | 202976_s_at | RHOBTB3 | Rho related BTB domain containing 3 |  | 201756_at | RPA2 | replication protein A2 |
|  | 203145_at | SPAG5 | sperm associated antigen 5 |  | 203877_at | - | - |
|  | 204134_at | PDE2A | phosphodiesterase 2A |  | 205582_s_at | GGT5 | gamma-glutamyltransferase 5 |
|  | 201698_s_at | GATC | glutamyl-tRNAamidotransferase subunit C |  | 200925_at | COX6A1 | cytochrome c oxidase subunit 6A1 |
|  | 208810_at | DNAJB6 | DnaJ heat shock protein family (Hsp40) member B6 |  | 201495_x_at | MYH11 | myosin heavy chain 11 |
|  | 200869_at | SNORA68 | small nucleolarRNA, H/ACA box 68 |  | 200981_x_at | GNAS | GNAS complex locus |
|  | 205765_at | CYP3A5 | cytochrome P450 family 3 subfamily A member 5 |  | 204530_s_at | TOX | thymocyte selection associated high mobility group box |
|  | 207657_x_at | TNPO1 | transportin 1 |  | 201062_at | STOM | stomatin |
|  | 203663_s_at | COX5A | cytochrome c oxidase subunit 5A |  | 206325_at | SERPINA6 | serpin family A member 6 |
|  | 203393_at | HES1 | hes family bHLH transcription factor 1 |  | 207166_at | GNGT1 | G protein subunit gamma transducin 1 |
|  | 202007_at | NID1 | nidogen 1 |  | 204486_at | KCNQ1OT1 | KCNQ1 opposite strand/antisense transcript 1 |
|  | 204565_at | ACOT13 | acyl-CoA thioesterase 13 |  | 203417_at | MFAP2 | microfibril associated protein 2 |
|  | 201326_at | CCT6A | chaperonin containing TCP1 subunit 6A |  | 203619_s_at | FAIM2 | Fas apoptotic inhibitory molecule 2 |
|  | 208755_x_at | H3P6 | H3 histone pseudogene 6 |  | 202765_s_at | FBN1 | fibrillin 1 |
|  | 200610_s_at | NCL | nucleolin |  | 201248_s_at | SREBF2 | sterol regulatory element binding transcription factor 2 |
|  | 208639_x_at | PDIA6 | protein disulfide isomerase family A member 6 |  | 207805_s_at | PSMD9 | proteasome 26S subunit, non-ATPase 9 |
|  | 207460_at | GZMM | granzyme M |  | 202639_s_at | RANBP3 | RAN binding protein 3 |
|  | 209055_s_at | CDC5L | cell division cycle 5 like |  | 204580_at | MMP12 | matrix metallopeptidase 12 |
|  | 205597_at | SLC44A4 | solute carrier family 44 member 4 |  | 205553_s_at | CSRP3 | cysteine and glycine rich protein 3 |
|  | 202376_at | SERPINA3 | serpin family A member 3 |  | 205097_at | SLC26A2 | solute carrier family 26 member 2 |
|  | 203895_at | PLCB4 | phospholipase C beta 4 |  | 203832_at | SNRPF | small nuclear ribonucleoprotein polypeptide F |
|  | 204226_at | STAU2 | staufen double-stranded RNA binding protein 2 |  | 207974_s_at | SKP1 | S-phase kinase associated protein 1 |
|  | 203842_s_at | MAPRE3 | microtubule associated protein RP/EB family member 3 |  | 202544_at | GMFB | glia maturation factor beta |
|  | 202309_at | MTHFD1 | methylenetetrahydrofolate dehydrogenase, cyclohydrolase and formyltetrahydrofolatesynthetase 1 |  | 207071_s_at | ACO1 | aconitase 1 |
|  | 201820_s_at | - | - |  | 206159_at | GDF10 | growth differentiation factor 10 |
|  | 206827_s_at | TRPV6 | transient receptor potential cation channel subfamily V member 6 |  | 205615_at | CPA1 | carboxypeptidase A1 |
|  | 200651_at | RACK1 | receptor for activated C kinase 1 |  | 207053_at | SLC8A1 | solute carrier family 8 member A1 |
|  | 206181_at | SLAMF1 | signaling lymphocytic activation molecule family member 1 |  | 206305_s_at | C8A | complement C8 alpha chain |
|  | 206102_at | GINS1 | GINS complex subunit 1 |  | 200807_s_at | HSPD1 | heat shock protein family D (Hsp60) member 1 |
|  | 203744_at | HMGB3 | high mobility group box 3 |  | 205967_at | H4C11 | H4 clustered histone 11 |
|  | 205544_s_at | CR2 | complement C3d receptor 2 |  | AFFX-PheX-3_at | - | - |
|  | 209114_at | TSPAN1 | tetraspanin 1 |  | 205078_at | PIGF | phosphatidylinositol glycan anchor biosynthesis class F |
|  | 204787_at | VSIG4 | V-set and immunoglobulin domain containing 4 |  | 201667_at | GJA1 | gap junction protein alpha 1 |
|  | 201283_s_at | TRAK1 | trafficking kinesin protein 1 |  | 205280_at | GLRB | glycine receptor beta |
|  | 201372_s_at | CUL3 | cullin 3 |  | 200928_s_at | RAB14 | RAB14, member RAS oncogene family |
|  | 208296_x_at | TNFAIP8 | TNF alpha induced protein 8 |  | 207513_s_at | ZNF189 | zinc finger protein 189 |
|  | 203477_at | COL15A1 | collagen type XV alpha 1 chain |  | 200657_at | SLC25A5 | solute carrier family 25 member 5 |
|  | 200603_at | PRKAR1A | protein kinase cAMP-dependent type I regulatory subunit alpha |  | 208706_s_at | EIF5 | eukaryotic translation initiation factor 5 |
|  | 206142_at | ZNF135 | zinc finger protein 135 |  | 201860_s_at | PLAT | plasminogen activator, tissue type |
|  | 202749_at | GET1 | guided entry of tail-anchored proteins factor 1 |  | 200685_at | SRSF11 | serine and arginine rich splicing factor 11 |
|  | 201610_at | ICMT | isoprenylcysteine carboxyl methyltransferase |  | 204854_at | P3H3 | prolyl 3-hydroxylase 3 |
|  | 204260_at | CHGB | chromogranin B |  | 206311_s_at | PLA2G1B | phospholipase A2 group IB |
|  | 207441_at | SMR3B | submaxillary gland androgen regulated protein 3B |  | 203675_at | NUCB2 | nucleobindin 2 |
|  | 204410_at | EIF1AY | eukaryotic translation initiation factor 1A Y-linked |  | 203716_s_at | DPP4 | dipeptidyl peptidase 4 |
|  | 208985_s_at | EIF3J | eukaryotic translation initiation factor 3 subunit J |  | 207618_s_at | BCS1L | BCS1 homolog, ubiquinol-cytochrome c reductase complex chaperone |
|  | 202196_s_at | DKK3 | dickkopf WNT signaling pathway inhibitor 3 |  | 209165_at | AATF | apoptosis antagonizing transcription factor |
|  | 205794_s_at | NOVA1 | NOVA alternative splicing regulator 1 |  | 202078_at | COPS3 | COP9 signalosome subunit 3 |
|  | 200939_s_at | RERE | arginine-glutamic acid dipeptide repeats |  | 204913_s_at | SOX11 | SRY-box transcription factor 11 |
|  | 209068_at | HNRNPDL | heterogeneous nuclear ribonucleoprotein D like |  | 208929_x_at | SNORD68 | small nucleolar RNA, C/D box 68 |
|  | 206952_at | G6PC | glucose-6-phosphatase catalytic subunit |  | 203621_at | NDUFB5 | NADH:ubiquinoneoxidoreductase subunit B5 |
|  | 200677_at | PTTG1IP | PTTG1 interacting protein |  | 201553_s_at | LAMP1 | lysosomal associated membrane protein 1 |
|  | 207616_s_at | TANK | TRAF family member associated NFKB activator |  | 203614_at | ALG11 | ALG11 alpha-1,2-mannosyltransferase |
|  | 201974_s_at | - | - |  | 204802_at | RRAD | RRAD, Ras related glycolysis inhibitor and calcium channel regulator |
|  | 201852_x_at | COL3A1 | collagen type III alpha 1 chain |  | 205982_x_at | SFTPC | surfactant protein C |
|  | 201451_x_at | RHEB | Ras homolog, mTORC1 binding |  | 202498_s_at | SLC2A3 | solute carrier family 2 member 3 |
|  | 206571_s_at | MAP4K4 | mitogen-activated protein kinase kinasekinasekinase 4 |  | 204304_s_at | PROM1 | prominin 1 |
|  | 201037_at | PFKP | phosphofructokinase, platelet |  | 206559_x_at | - | - |
|  | 205443_at | SNAPC1 | small nuclear RNA activating complex polypeptide 1 |  | 201550_x_at | ACTG1 | actin gamma 1 |
|  | 202652_at | APBB1 | amyloid beta precursor protein binding family B member 1 |  | 203697_at | FRZB | frizzled related protein |
|  | 205644_s_at | SNRPG | small nuclear ribonucleoprotein polypeptide G |  | 205936_s_at | HK3 | hexokinase 3 |
|  | 205618_at | PRRG1 | proline rich and Gla domain 1 |  | 201665_x_at | RPS17 | ribosomal protein S17 |
|  | 206013_s_at | ACTL6B | actin like 6B |  | 206193_s_at | CDSN | corneodesmosin |
|  | 207124_s_at | GNB5 | G protein subunit beta 5 |  | 207766_at | CDKL1 | cyclin dependent kinase like 1 |
|  | 200077_s_at | OAZ1 | ornithine decarboxylase antizyme 1 |  | 204258_at | CHD1 | chromodomain helicase DNA binding protein 1 |
|  | 202887_s_at | DDIT4 | DNA damage inducible transcript 4 |  | 203737_s_at | PPRC1 | PPARG related coactivator 1 |
|  | 206475_x_at | CSHL1 | chorionic somatomammotropin hormone like 1 |  | 201069_at | MMP2 | matrix metallopeptidase 2 |
|  | 200997_at | RBM4 | RNA binding motif protein 4 |  | 204170_s_at | CKS2 | CDC28 protein kinase regulatory subunit 2 |
|  | 202928_s_at | PHF1 | PHD finger protein 1 |  | 206156_at | GJB5 | gap junction protein beta 5 |
|  | 202317_s_at | UBE4B | ubiquitination factor E4B |  | 201789_at | PCNX4 | pecanex 4 |
|  | 203276_at | LMNB1 | lamin B1 |  | 208072_s_at | DGKD | diacylglycerol kinase delta |
|  | 201160_s_at | YBX3 | Y-box binding protein 3 |  | Affx_Trpnx_M_at | - | - |
|  | 205343_at | SULT1C2 | sulfotransferase family 1C member 2 |  | 202465_at | PCOLCE | procollagen C-endopeptidase enhancer |
|  | 207707_s_at | SEC13 | SEC13 homolog, nuclear pore and COPII coat complex component |  | 206322_at | SYN3 | synapsin III |
|  | 204414_at | LARGE1 | LARGE xylosyl- and glucuronyltransferase 1 |  | 202769_at | CCNG2 | cyclin G2 |
|  | 205711_x_at | ATP5F1C | ATP synthase F1 subunit gamma |  | 200937_s_at | RPL5 | ribosomal protein L5 |
|  | 206230_at | LHX1 | LIM homeobox 1 |  | 204874_x_at | BAIAP3 | BAI1 associated protein 3 |
|  | 206330_s_at | SHC3 | SHC adaptor protein 3 |  | 202850_at | ABCD3 | ATP binding cassette subfamily D member 3 |
|  | 204450_x_at | APOA1 | apolipoprotein A1 |  | 206067_s_at | WT1 | WT1 transcription factor |
|  | 202892_at | CDC23 | cell division cycle 23 |  | 204228_at | PPIH | peptidylprolylisomerase H |
|  | 203215_s_at | MYO6 | myosin VI |  | 200087_s_at | TMED2 | transmembrane p24 trafficking protein 2 |
|  | 205891_at | ADORA2B | adenosine A2b receptor) |  | 203789_s_at | SEMA3C | semaphorin 3C |
|  | 200650_s_at | LDHA | lactate dehydrogenase A |  | 204560_at | FKBP5 | FKBP prolylisomerase 5 |
|  | 205714_s_at | ZMYND10 | zinc finger MYND-type containing 10 |  | 202016_at | MEST | mesoderm specific transcript |
|  | 202854_at | HPRT1 | hypoxanthine phosphoribosyltransferase 1 |  | 205559_s_at | PCSK5 | proproteinconvertasesubtilisin/kexin type 5 |
|  | 205767_at | EREG | epiregulin |  | 209200_at | MEF2C | myocyte enhancer factor 2C |
|  | 201975_at | CLIP1 | CAP-Gly domain containing linker protein 1 |  | 206295_at | IL18 | interleukin 18 |
|  | 203633_at | CPT1A | carnitinepalmitoyltransferase 1A |  | 201916_s_at | SEC63 | SEC63 homolog, protein translocation regulator |
|  | 202951_at | STK38 | serine/threonine kinase 38 |  | 208888_s_at | NCOR2 | nuclear receptor corepressor 2 |
|  | 205030_at | FABP7 | fatty acid binding protein 7 |  | 203408_s_at | SATB1 | SATB homeobox 1 |
|  | 205724_at | PKP1 | plakophilin 1 |  | 205651_x_at | RAPGEF4 | Rap guanine nucleotide exchange factor 4 |
|  | 204416_x_at | APOC1 | apolipoprotein C1 |  | 205659_at | HDAC9 | histone deacetylase 9 |
|  | 205113_at | NEFM | neurofilament medium |  | 205373_at | CTNNA2 | catenin alpha 2 |
|  | 207147_at | DLX2 | distal-less homeobox 2 |  | 202284_s_at | CDKN1A | cyclin dependent kinase inhibitor 1A |
