# Supplementary material for: Unraveling the multi-targeted curative potential of bioactive molecules against cervical cancer through integrated omics and systems pharmacology approach
Source: Sci Rep. 2022 Aug 21;12:14245. doi: 10.1038/s41598-022-18358-7 (PMC9393168; doi:10.1038/s41598-022-18358-7)
Supplement: Supplementary file 3 — Supplementary Table S2. [file 41598_2022_18358_MOESM3_ESM.docx]

Supplementary Table S2: List of genes identified to be Up and **Down Regulated**

| **No** | **Probe Set ID** | **Gene Symbol** | **Differential Expression** | **No** | **Probe Set ID** | **Gene Symbol** | **Differential Expression** |
| --- | --- | --- | --- | --- | --- | --- | --- |
|  | 208805_at | - | **Down Regulated** |  | 200819_s_at | RPS15 | **Down Regulated** |
|  | 206790_s_at | NDUFB1 | **Down Regulated** |  | 208581_x_at | MT1X | **Down Regulated** |
|  | 204252_at | CDK2 |  |  | 208628_s_at | YBX1 | **Down Regulated** |
|  | 208780_x_at | VAPA | **Down Regulated** |  | 208865_at | CSNK1A1 | **Down Regulated** |
|  | 208855_s_at | STK24 | **Down Regulated** |  | 207573_x_at | ATP5MG | **Down Regulated** |
|  | 207828_s_at | CENPF | **Down Regulated** |  | 208786_s_at | MAP1LC3B | **Down Regulated** |
|  | 200673_at | LAPTM4A | **Down Regulated** |  | 200626_s_at | MATR3 | **Down Regulated** |
|  | 205819_at | MARCO |  |  | 202532_s_at | DHFR | **Down Regulated** |
|  | 201726_at | ELAVL1 | **Down Regulated** |  | 200647_x_at | EIF3C |  |
|  | 208078_s_at | SIK1 | **Down Regulated** |  | 207040_s_at | ST13 | **Down Regulated** |
|  | 202252_at | RAB13 | **Down Regulated** |  | 201744_s_at | LUM |  |
|  | 208834_x_at | RPL23A | **Down Regulated** |  | 209024_s_at | SYNCRIP | **Down Regulated** |
|  | 202707_at | UMPS | Up regulated |  | 205055_at | ITGAE | **Down Regulated** |
|  | 205503_at | PTPN14 | Up regulated |  | 206100_at | CPM | **Down Regulated** |
|  | 204704_s_at | ALDOB | Up regulated |  | 207126_x_at | UGT1A10 | **Down Regulated** |
|  | 208029_s_at | LAPTM4B | **Down Regulated** |  | 200079_s_at | KARS1 | **Down Regulated** |
|  | 203673_at | TG | Up regulated |  | 206662_at | GLRX | **Down Regulated** |
|  | 206659_at | - | Up regulated |  | 205721_at | GFRA2 | Up regulated |
|  | 200614_at | CLTC | Up regulated |  | 200628_s_at | WARS1 | **Down Regulated** |
|  | 207243_s_at | CALM3 | **Down Regulated** |  | 208905_at | CYCS | **Down Regulated** |
|  | 205056_s_at | GPR162 | Up regulated |  | 205157_s_at | KRT17 | **Down Regulated** |
|  | 203924_at | GSTA1 | Up regulated |  | 203591_s_at | CSF3R | Up regulated |
|  | 205390_s_at | ANK1 | Up regulated |  | 201428_at | CLDN4 | Up regulated |
|  | 208904_s_at | RPS28 | **Down Regulated** |  | 206055_s_at | SNRPA1 | **Down Regulated** |
|  | 209007_s_at | RSRP1 | **Down Regulated** |  | 205733_at | BLM | **Down Regulated** |
|  | 204273_at | EDNRB | Up regulated |  | 204551_s_at | AHSG | Up regulated |
|  | 206597_at | NRL | Up regulated |  | 204528_s_at | NAP1L1 | **Down Regulated** |
|  | 200624_s_at | SNHG4 | **Down Regulated** |  | 205358_at | GRIA2 | Up regulated |
|  | 201878_at | ARIH1 | **Down Regulated** |  | 200876_s_at | PSMB1 | **Down Regulated** |
|  | 208152_s_at | DDX21 | Up regulated |  | 208659_at | CLIC1 | **Down Regulated** |
|  | 200099_s_at | RPS3A | **Down Regulated** |  | 201682_at | PMPCB | **Down Regulated** |
|  | 205124_at | BORCS8 | Up regulated |  | 201981_at | PAPPA | Up regulated |
|  | 205860_x_at | FOLH1B | Up regulated |  | 208093_s_at | NDEL1 | **Down Regulated** |
|  | 207074_s_at | SLC18A1 | Up regulated |  | 201427_s_at | SELENOP | Up regulated |
|  | 202287_s_at | TACSTD2 | Up regulated |  | 208612_at | PDIA3 | **Down Regulated** |
|  | 208270_s_at | RNPEP | **Down Regulated** |  | 203566_s_at | AGL | **Down Regulated** |
|  | 207988_s_at | ARPC2 | **Down Regulated** |  | 207076_s_at | ASS1 | **Down Regulated** |
|  | 204992_s_at | PFN2 | Up regulated |  | 208887_at | EIF3G | **Down Regulated** |
|  | 206094_x_at | UGT1A5 | **Down Regulated** |  | 202011_at | TJP1 | **Down Regulated** |
|  | 205555_s_at | MSX2 | Up regulated |  | 201141_at | GPNMB | Up regulated |
|  | 202158_s_at | CELF2 | Up regulated |  | 205985_x_at | CLCNKB | Up regulated |
|  | 208926_at | NEU1 | **Down Regulated** |  | 200721_s_at | ACTR1A | Up regulated |
|  | 203917_at | CXADR | **Down Regulated** |  | 209097_s_at | JAG1 | Up regulated |
|  | 202746_at | ITM2A | Up regulated |  | 204086_at | PRAME | Up regulated |
|  | 201468_s_at | NQO1 | **Down Regulated** |  | 207588_at | - | Up regulated |
|  | 202298_at | NDUFA1 | **Down Regulated** |  | 202833_s_at | SERPINA1 | Up regulated |
|  | 206874_s_at | SLK | **Down Regulated** |  | 200639_s_at | YWHAZ | **Down Regulated** |
|  | 203967_at | CDC6 | **Down Regulated** |  | 202581_at | HSPA1B | Up regulated |
|  | 203233_at | IL4R | Up regulated |  | 204550_x_at | GSTM1 | Up regulated |
|  | 203804_s_at | LUC7L3 | **Down Regulated** |  | 205583_s_at | ALG13 | Up regulated |
|  | 201802_at | SLC29A1 | Up regulated |  | 200640_at | YWHAZ | **Down Regulated** |
|  | 203750_s_at | RARA | Up regulated |  | 204868_at | MRPL58 | **Down Regulated** |
|  | 200084_at | C11orf58 | **Down Regulated** |  | 202521_at | CTCF | **Down Regulated** |
|  | 204173_at | MYL6B | **Down Regulated** |  | 201570_at | SAMM50 | **Down Regulated** |
|  | 200641_s_at | YWHAZ | **Down Regulated** |  | 204850_s_at | DCX | Up regulated |
|  | 203568_s_at | TRIM38 | **Down Regulated** |  | 202711_at | EFNB1 | Up regulated |
|  | 201951_at | ALCAM | Up regulated |  | 202146_at | IFRD1 | **Down Regulated** |
|  | 202436_s_at | CYP1B1 | **Down Regulated** |  | 200785_s_at | LRP1 | Up regulated |
|  | 202302_s_at | RSRC2 | **Down Regulated** |  | 204192_at | CD37 | Up regulated |
|  | 201324_at | EMP1 | **Down Regulated** |  | 207140_at | ALPI | Up regulated |
|  | 203443_at | EML3 | Up regulated |  | 204581_at | CD22 | Up regulated |
|  | 202111_at | SLC4A2 | Up regulated |  | 205286_at | TFAP2C | **Down Regulated** |
|  | 202154_x_at | TUBB3 | **Down Regulated** |  | 203042_at | LAMP2 | **Down Regulated** |
|  | 206461_x_at | MT1H | **Down Regulated** |  | 202992_at | C7 | Up regulated |
|  | 202284_s_at | CDKN1A | Up regulated |  | 205523_at | HAPLN1 | Up regulated |
|  | 203465_at | MRPL19 | **Down Regulated** |  | 200986_at | SERPING1 | Up regulated |
|  | 205686_s_at | CD86 | Up regulated |  | 205005_s_at | NMT2 | **Down Regulated** |
|  | 204960_at | PTPRCAP | Up regulated |  | 202664_at | WIPF1 | Up regulated |
|  | 211540_s_at | RB1 | Up regulated |  | 206840_at | AFM | Up regulated |
|  | 202470_s_at | CPSF6 | **Down Regulated** |  | 208815_x_at | HSPA4 | **Down Regulated** |
|  | 204163_at | EMILIN1 | Up regulated |  | 200707_s_at |  | Up regulated |
|  | 200034_s_at | RPL6 | **Down Regulated** |  | 200796_s_at | MCL1 | Up regulated |
|  | 206387_at | CDX2 | Up regulated |  | 202517_at | CRMP1 | Up regulated |
|  | 202988_s_at | RGS1 | Up regulated |  | 200917_s_at | SRPRA | **Down Regulated** |
|  | 204151_x_at | - | **Down Regulated** |  | 201318_s_at | MYL12B | **Down Regulated** |
|  | 207508_at | ATP5MC3 | **Down Regulated** |  | 202440_s_at | DENND2B | Up regulated |
|  | 200951_s_at | CCND2 | Up regulated |  | 204712_at | WIF1 | Up regulated |
|  | 206646_at | GLI1 | Up regulated |  | 202902_s_at | CTSS | Up regulated |
|  | 207030_s_at | CSRP2 | **Down Regulated** |  | 205909_at | POLE2 | **Down Regulated** |
|  | 204389_at | MAOA | Up regulated |  | 202989_at | RGS1 | Up regulated |
|  | 204910_s_at | TRIM3 | Up regulated |  | 200711_s_at | SKP1 | **Down Regulated** |
|  | 206291_at | NTS | Up regulated |  | 204905_s_at | EEF1E1 | Up regulated |
|  | 204046_at | PLCB2 | Up regulated |  | 205822_s_at | HMGCS1 | **Down Regulated** |
|  | 203896_s_at | PLCB4 | Up regulated |  | 208617_s_at | PTP4A2 | **Down Regulated** |
|  | 203416_at | CD53 | Up regulated |  | RARA | PARP1 | Up regulated |
|  | 205907_s_at | OMD | Up regulated |  | 209205_s_at | LMO4 | **Down Regulated** |
|  | 203998_s_at | SYT1 | Up regulated |  | 200071_at | SMNDC1 | **Down Regulated** |
|  | 208097_s_at | TMX1 | **Down Regulated** |  | 203034_s_at | RPL27A | **Down Regulated** |
|  | 202620_s_at | PLOD2 | **Down Regulated** |  | 201265_at | - | Up regulated |
|  | 204659_s_at | GFER | **Down Regulated** |  | 203424_s_at | IGFBP5 | Up regulated |
|  | 204744_s_at | IARS1 | **Down Regulated** |  | 205312_at | SPI1 | Up regulated |
|  | 205543_at | HSPA4L | **Down Regulated** |  | 203589_s_at | TFDP2 | **Down Regulated** |
|  | 205719_s_at | PAH | Up regulated |  | 205483_s_at | ISG15 | **Down Regulated** |
|  | 208528_x_at | SSX7 | Up regulated |  | 205649_s_at | FGA | Up regulated |
|  | 204092_s_at | AURKA | **Down Regulated** |  | 204417_at | GALC | **Down Regulated** |
|  | 202115_s_at | NOC2L | **Down Regulated** |  | 208720_s_at | RBM39 | **Down Regulated** |
|  | 203993_x_at | CFAP410 | Up regulated |  | 201654_s_at | HSPG2 | Up regulated |
|  | 202210_x_at | GSK3A | Up regulated |  | 202002_at | ACAA2 | Up regulated |
|  | 205812_s_at | TMED9 | **Down Regulated** |  | 205152_at | SLC6A1 | Up regulated |
|  | 202976_s_at | RHOBTB3 | **Down Regulated** |  | 201756_at | RPA2 | **Down Regulated** |
|  | 203145_at | SPAG5 | **Down Regulated** |  | 203877_at | - | Up regulated |
|  | 204134_at | PDE2A | Up regulated |  | 205582_s_at | GGT5 | Up regulated |
|  | 201698_s_at | GATC | **Down Regulated** |  | 200925_at | COX6A1 | **Down Regulated** |
|  | 208810_at | DNAJB6 | **Down Regulated** |  | 201495_x_at | MYH11 | Up regulated |
|  | 200869_at | SNORA68 | **Down Regulated** |  | 200981_x_at | GNAS | **Down Regulated** |
|  | 205765_at | CYP3A5 | Up regulated |  | 204530_s_at | TOX | Up regulated |
|  | 207657_x_at | TNPO1 | **Down Regulated** |  | 201062_at | STOM | Up regulated |
|  | 203663_s_at | COX5A | **Down Regulated** |  | 206325_at | SERPINA6 | Up regulated |
|  | 203393_at | HES1 | Up regulated |  | 207166_at | GNGT1 | Up regulated |
|  | 202007_at | NID1 | Up regulated |  | 204486_at | KCNQ1OT1 | Up regulated |
|  | 204565_at | ACOT13 | **Down Regulated** |  | 203417_at | MFAP2 | Up regulated |
|  | 201326_at | CCT6A | **Down Regulated** |  | 203619_s_at | FAIM2 | Up regulated |
|  | 208755_x_at | H3P6 | **Down Regulated** |  | 202765_s_at | FBN1 | Up regulated |
|  | 200610_s_at | NCL | **Down Regulated** |  | 201248_s_at | SREBF2 | Up regulated |
|  | 208639_x_at | PDIA6 | **Down Regulated** |  | 207805_s_at | PSMD9 | **Down Regulated** |
|  | 207460_at | GZMM | Up regulated |  | 202639_s_at | RANBP3 | Up regulated |
|  | 209055_s_at | CDC5L | **Down Regulated** |  | 204580_at | MMP12 | Up regulated |
|  | 205597_at | SLC44A4 | Up regulated |  | 205553_s_at | CSRP3 | Up regulated |
|  | 202376_at | SERPINA3 | Up regulated |  | 205097_at | SLC26A2 | **Down Regulated** |
|  | 203895_at | PLCB4 | Up regulated |  | 203832_at | SNRPF | **Down Regulated** |
|  | 204226_at | STAU2 | **Down Regulated** |  | 207974_s_at | SKP1 | **Down Regulated** |
|  | 203842_s_at | MAPRE3 | Up regulated |  | 202544_at | GMFB | **Down Regulated** |
|  | 202309_at | MTHFD1 | **Down Regulated** |  | 207071_s_at | ACO1 | **Down Regulated** |
|  | 201820_s_at | - | Up regulated |  | 206159_at | GDF10 | Up regulated |
|  | 206827_s_at | TRPV6 | Up regulated |  | 205615_at | CPA1 | Up regulated |
|  | 200651_at | RACK1 | Up regulated |  | 207053_at | SLC8A1 | Up regulated |
|  | 206181_at | SLAMF1 | Up regulated |  | 206305_s_at | C8A | Up regulated |
|  | 206102_at | GINS1 | **Down Regulated** |  | 200807_s_at | HSPD1 | **Down Regulated** |
|  | 203744_at | HMGB3 | **Down Regulated** |  | 205967_at | H4C11 | **Down Regulated** |
|  | 205544_s_at | CR2 | Up regulated |  | AFFX-PheX-3_at | - | Up regulated |
|  | 209114_at | TSPAN1 | Up regulated |  | 205078_at | PIGF | **Down Regulated** |
|  | 204787_at | VSIG4 | Up regulated |  | 201667_at | GJA1 | Up regulated |
|  | 201283_s_at | TRAK1 | Up regulated |  | 205280_at | GLRB | Up regulated |
|  | 201372_s_at | CUL3 | Up regulated |  | 200928_s_at | RAB14 | Up regulated |
|  | 208296_x_at | TNFAIP8 | **Down Regulated** |  | 207513_s_at | ZNF189 | **Down Regulated** |
|  | 203477_at | COL15A1 | Up regulated |  | 200657_at | SLC25A5 | **Down Regulated** |
|  | 200603_at | PRKAR1A | **Down Regulated** |  | 208706_s_at | EIF5 | **Down Regulated** |
|  | 206142_at | ZNF135 | Up regulated |  | 201860_s_at | PLAT | Up regulated |
|  | 202749_at | GET1 | Up regulated |  | 200685_at | SRSF11 | Up regulated |
|  | 201610_at | ICMT | Up regulated |  | 204854_at | P3H3 | Up regulated |
|  | 204260_at | CHGB | Up regulated |  | 206311_s_at | PLA2G1B | Up regulated |
|  | 207441_at | SMR3B | Up regulated |  | 203675_at | NUCB2 | **Down Regulated** |
|  | 204410_at | EIF1AY | Up regulated |  | 203716_s_at | DPP4 | Up regulated |
|  | 208985_s_at | EIF3J | **Down Regulated** |  | 207618_s_at | BCS1L | **Down Regulated** |
|  | 202196_s_at | DKK3 | Up regulated |  | 209165_at | AATF | **Down Regulated** |
|  | 205794_s_at | NOVA1 | Up regulated |  | 202078_at | COPS3 | **Down Regulated** |
|  | 200939_s_at | RERE | Up regulated |  | 204913_s_at | SOX11 | Up regulated |
|  | 209068_at | HNRNPDL | Up regulated |  | 208929_x_at | SNORD68 | **Down Regulated** |
|  | 206952_at | G6PC | Up regulated |  | 203621_at | NDUFB5 | **Down Regulated** |
|  | 200677_at | PTTG1IP | **Down Regulated** |  | 201553_s_at | LAMP1 | **Down Regulated** |
|  | 207616_s_at | TANK | Up regulated |  | 203614_at | ALG11 | **Down Regulated** |
|  | 201974_s_at | - | Up regulated |  | 204802_at | RRAD | Up regulated |
|  | 201852_x_at | COL3A1 | Up regulated |  | 205982_x_at | SFTPC | Up regulated |
|  | 201451_x_at | RHEB | Up regulated |  | 202498_s_at | SLC2A3 | Up regulated |
|  | 206571_s_at | MAP4K4 | **Down Regulated** |  | 204304_s_at | PROM1 | Up regulated |
|  | 201037_at | PFKP | **Down Regulated** |  | 206559_x_at | - | **Down Regulated** |
|  | 205443_at | SNAPC1 | **Down Regulated** |  | 201550_x_at | ACTG1 | **Down Regulated** |
|  | 202652_at | APBB1 | Up regulated |  | 203697_at | FRZB | Up regulated |
|  | 205644_s_at | SNRPG | **Down Regulated** |  | 205936_s_at | HK3 | Up regulated |
|  | 205618_at | PRRG1 | **Down Regulated** |  | 201665_x_at | RPS17 | **Down Regulated** |
|  | 206013_s_at | ACTL6B | Up regulated |  | 206193_s_at | CDSN | Up regulated |
|  | 207124_s_at | GNB5 | **Down Regulated** |  | 207766_at | CDKL1 | Up regulated |
|  | 200077_s_at | OAZ1 | **Down Regulated** |  | 204258_at | CHD1 | **Down Regulated** |
|  | 202887_s_at | DDIT4 | **Down Regulated** |  | 203737_s_at | PPRC1 | **Down Regulated** |
|  | 206475_x_at | CSHL1 | **Down Regulated** |  | 201069_at | MMP2 | Up regulated |
|  | 200997_at | RBM4 | **Down Regulated** |  | 204170_s_at | CKS2 | Up regulated |
|  | 202928_s_at | PHF1 | Up regulated |  | 206156_at | GJB5 | Up regulated |
|  | 202317_s_at | UBE4B | **Down Regulated** |  | 201789_at | PCNX4 | Up regulated |
|  | 203276_at | LMNB1 | **Down Regulated** |  | 208072_s_at | DGKD | **Down Regulated** |
|  | 201160_s_at | YBX3 | **Down Regulated** |  | Affx_Trpnx_M_at | - | Up regulated |
|  | 205343_at | SULT1C2 | Up regulated |  | 202465_at | PCOLCE | Up regulated |
|  | 207707_s_at | SEC13 | **Down Regulated** |  | 206322_at | SYN3 | Up regulated |
|  | 204414_at | LARGE1 | Up regulated |  | 202769_at | CCNG2 | **Down Regulated** |
|  | 205711_x_at | ATP5F1C | **Down Regulated** |  | 200937_s_at | RPL5 | **Down Regulated** |
|  | 206230_at | LHX1 | Up regulated |  | 204874_x_at | BAIAP3 | Up regulated |
|  | 206330_s_at | SHC3 | Up regulated |  | 202850_at | ABCD3 | **Down Regulated** |
|  | 204450_x_at | APOA1 | Up regulated |  | 206067_s_at | WT1 | Up regulated |
|  | 202892_at | CDC23 | **Down Regulated** |  | 204228_at | PPIH | **Down Regulated** |
|  | 203215_s_at | MYO6 | Up regulated |  | 200087_s_at | TMED2 | **Down Regulated** |
|  | 205891_at | ADORA2B | **Down Regulated** |  | 203789_s_at | SEMA3C | **Down Regulated** |
|  | 200650_s_at | LDHA | **Down Regulated** |  | 204560_at | FKBP5 | Up regulated |
|  | 205714_s_at | ZMYND10 | Up regulated |  | 202016_at | MEST | **Down Regulated** |
|  | 202854_at | HPRT1 | **Down Regulated** |  | 205559_s_at | PCSK5 | **Down Regulated** |
|  | 205767_at | EREG | **Down Regulated** |  | 209200_at | MEF2C |  |
|  | 201975_at | CLIP1 | **Down Regulated** |  | 206295_at | IL18 | **Down Regulated** |
|  | 203633_at | CPT1A | **Down Regulated** |  | 201916_s_at | SEC63 | **Down Regulated** |
|  | 202951_at | STK38 | **Down Regulated** |  | 208888_s_at | NCOR2 | Up regulated |
|  | 205030_at | FABP7 | Up regulated |  | 203408_s_at | SATB1 | Up regulated |
|  | 205724_at | PKP1 | Up regulated |  | 205651_x_at | RAPGEF4 | Up regulated |
|  | 204416_x_at | APOC1 | Up regulated |  | 205659_at | HDAC9 | Up regulated |
|  | 205113_at | NEFM | Up regulated |  | 205373_at | CTNNA2 | Up regulated |
|  | 207147_at | DLX2 | **Down Regulated** |  | 202284_s_at | CDKN1A | Up regulated |
